# [^11^C]MODAG-001—towards a PET tracer targeting α-synuclein aggregates

**DOI:** 10.1007/s00259-020-05133-x

**Published:** 2020-12-28

**Authors:** Laura Kuebler, Sabrina Buss, Andrei Leonov, Sergey Ryazanov, Felix Schmidt, Andreas Maurer, Daniel Weckbecker, Anne M. Landau, Thea P. Lillethorup, Daniel Bleher, Ran Sing Saw, Bernd J. Pichler, Christian Griesinger, Armin Giese, Kristina Herfert

**Affiliations:** 1 Werner Siemens Imaging Center, Department of Preclinical Imaging and Radiopharmacy, Eberhard Karls University of Tübingen, Röntgenweg 13, 72076 Tübingen, Germany; 2 MODAG GmbH, Mikroforum Ring 3, 55234 Wendelsheim, Germany; 3 Department of NMR-based Structural Biology, Max Planck Institute for Biophysical Chemistry, Am Faßberg 11, 37077 Göttingen, Germany; 4 Translational Neuropsychiatry Unit, Aarhus University, Norrebrogade 44, 8000 Aarhus, Denmark; 5 Department of Nuclear Medicine and PET-Centre, Aarhus University, Palle Juul-Jensens 165, J109, 8200 Aarhus, Denmark; 6 University Göttingen, Cluster of Excellence Multiscale Bioimaging Molecular Machines, 37077 Göttingen, Germany

**Keywords:** Alpha-synuclein, Parkinson’s disease, PET imaging, Tracer development

## Abstract

**Purpose:**

Deposition of misfolded alpha-synuclein (αSYN) aggregates in the human brain is one of the major hallmarks of synucleinopathies. However, a target-specific tracer to detect pathological aggregates of αSYN remains lacking. Here, we report the development of a positron emission tomography (PET) tracer based on anle138b, a compound shown to have therapeutic activity in animal models of neurodegenerative diseases.

**Methods:**

Specificity and selectivity of [^3^H]MODAG-001 were tested in in vitro binding assays using recombinant fibrils. After carbon-11 radiolabeling, the pharmacokinetic and metabolic profile was determined in mice. Specific binding was quantified in rats, inoculated with αSYN fibrils and using in vitro autoradiography in human brain sections of Lewy body dementia (LBD) cases provided by the Neurobiobank Munich (NBM).

**Results:**

[^3^H]MODAG-001 revealed a very high affinity towards pure αSYN fibrils (*K*_d_ = 0.6 ± 0.1 nM) and only a moderate affinity to hTau46 fibrils (*K*_d_ = 19 ± 6.4 nM) as well as amyloid-β_1–42_ fibrils (*K*_d_ = 20 ± 10 nM). [^11^C]MODAG-001 showed an excellent ability to penetrate the mouse brain. Metabolic degradation was present, but the stability of the parent compound improved after selective deuteration of the precursor. (d_3_)-[^11^C]MODAG-001 binding was confirmed in fibril-inoculated rat striata using in vivo PET imaging. In vitro autoradiography showed no detectable binding to aggregated αSYN in human brain sections of LBD cases, most likely, because of the low abundance of aggregated αSYN against background protein.

**Conclusion:**

MODAG-001 provides a promising lead structure for future compound development as it combines a high affinity and good selectivity in fibril-binding assays with suitable pharmacokinetics and biodistribution properties.

**Supplementary Information:**

The online version contains supplementary material available at 10.1007/s00259-020-05133-x.

## Introduction

The molecular pathogenesis of all common neurodegenerative diseases is associated with the disease-specific aggregation of misfolded proteins. Aggregation of α-synuclein (αSYN) has been shown to play a crucial role in the pathogenesis of Parkinson’s disease (PD) [1], Lewy body dementia (LBD), and multiple system atrophy (MSA) [2, 3]. In PD and LBD [4, 5], αSYN-containing inclusions accumulate in Lewy bodies (LBs) [6] and Lewy neurites (LNs).

Noninvasive imaging technologies such as magnetic resonance imaging (MRI) and positron emission tomography (PET) could serve as a valuable tool for the early and differential diagnosis of neurodegenerative diseases. Furthermore, they would allow for the monitoring of disease progression as well as evaluation of disease-modifying therapies. Whereas PET imaging of amyloid-β (Aβ) in Alzheimer’s disease (AD) is well established [7–9], PET tracers to detect pathologically aggregated αSYN in synucleinopathies are still missing despite intensive research.

Challenges complicating their identification might be (i) the target structure, as pathological αSYN might be present mainly in an oligomeric form rather than as fibrillar structures; (ii) the target concentration, as the amount of aggregated protein in condensed fibrillar αSYN aggregates (LBs, LNs) are by far less abundant than, for example, Aβ plaques; (iii) the target localization, as pathological αSYN is located intracellularly, making the cell membrane an additional physical barrier for tracer accessibility; (iv) the target composition, as β-sheet-binding motifs might be shared between different pathological protein aggregates, therefore complicating differential diagnosis or leading to unselective binding in the case of common co-pathologies; (v) the highly complex LB and LN composition comprising lipid membrane fragments and distorted organelles as recently shown by [10], which may hamper binding to αSYN fibrils; and (vi) the tracer structure, as lipophilic compounds that efficiently cross the blood-brain barrier (BBB) might show high nonspecific binding, reducing the signal-to-noise ratio (SNR) [11–13].

**Fig. 1:**
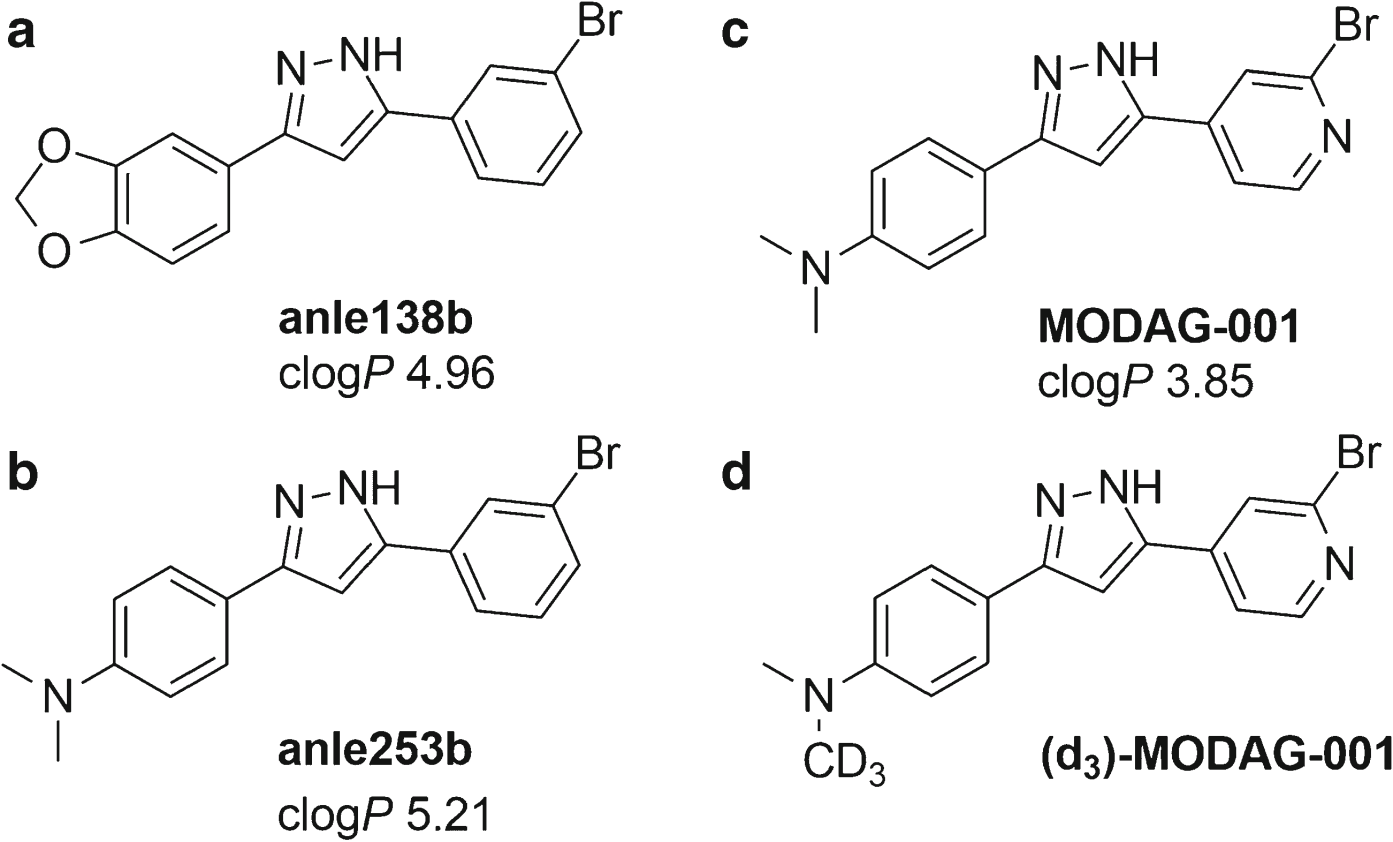
Chemical structures and calculated log*P* values (clog*P*) of anle138b (**a**), anle253b (**b**) MODAG-001 (**c**), and (d_3_)-MODAG-001 (**d**) [14]

Here, we aimed to develop a PET tracer starting from the lead structure anle138b (Fig. 1a), a compound that has been shown to have therapeutic activity in animal models of PD [15, 16] and MSA [17] based on specific structure-dependent binding to aggregated αSYN [18]. A previous derivative of anle138b, anle253b (Fig. 1b), was suited for ^11^C-labeling and showed high affinity towards αSYN aggregates in vitro; however, it showed suboptimal pharmacokinetics in vivo [14]. Therefore, we further modified the chemical structure of anle253b by exchanging the bromophenyl moiety with bromopyridine to reduce lipophilicity and improve the pharmacokinetic properties while keeping or even improving binding affinity (Fig. 1c). The resulting compound—MODAG-001—was evaluated in the current study using in vitro binding experiments on recombinant fibrils; autoradiography (AR) of human brain tissue with confirmed αSYN, Tau, and Aβ pathology [19]; pharmacokinetic analysis in vivo using mice; and the detection of stereotactically inoculated αSYN aggregates in vivo in a rat model.

## Material and methods

### Precursor and standard synthesis of MODAG-001 and (d_3_)-MODAG-001

The synthesis of MODAG-001, (d_3_)-MODAG-001, and the respective precursors for radiosynthesis is described in the supplementary material and methods section. The chemical structures are shown in Supplemental Fig. S1.

### MODAG-001 tritiation

MODAG-001 (Fig. 1c) was tritiated (RC Tritec AG, Teufen Switzerland), dissolved in ethanol and stored at − 80 °C until further usage. Its molar activity (A_m_) was 2.6 GBq/μmol, and its radiochemical purity was > 99% determined by high-performance liquid chromatography (HPLC).

### Fibril-binding experiments

Preparation and characterization of recombinant αSYN, hTau46, and Aβ_1–42_ fibrils are described in the supplementary material and methods section.

For saturation binding assays, the optimal fibril concentrations were determined according to Auld et al. [20] using a concentration determination assay (see supplementary material and methods section). Sonicated human recombinant αSYN (15 nM), hTau46 (250 nM), and Aβ_1–42_ (1 μM) fibrils diluted in phosphate-buffered saline (PBS) were incubated in low-binding plates (96-well micro test plate, Ratiolab GmbH, Dreieich, Germany) with [^3^H]MODAG-001 at increasing concentrations (0.05–12 nM/24 nM) in 30 mM Tris-HCl, 10% ethanol, 0.05% Tween20, pH 7.4 in a total volume of 200 μL/well. Nonspecific binding of the radiotracer was determined by coincubation with 400 nM nonlabeled MODAG-001 dissolved in DMSO (final DMSO concentration 0.04%). To determine the potential binding site of MODAG-001, a competition binding assay using SIL26 previously evaluated by Bagchi et al. [24] was performed. Recombinant αSYN fibrils (sonicated, 15 nM/) were incubated with 1 nM [^3^H]MODAG**-**001 and decreasing concentrations of a 1:4 serial dilution of nonlabeled SIL26 (4 μM**-**0.06 nM) in 30 mM Tris-HCl, 10% ethanol, 0.05% Tween20, pH 7.4 in a total volume of 200 μL/well.

Filtration and readout of competition and saturation binding experiments were performed as described in the supplementary material and methods section.

### Sensitivity analysis in mouse brain homogenates

To determine the limit of detection of [^3^H]MODAG-001 for αSYN fibrils in brain homogenates, one C57BL/6 mouse was sacrificed with CO_2_, and its brain was surgically removed. PBS containing one tablet each of phosphatase inhibitors (PhosSTOP™ phosphatase inhibitor tablets, Hoffmann-La Roche, Basel, Switzerland) and proteinase inhibitors (cOmplete™ protease inhibitor cocktail, Hoffmann-La Roche) per 5 mL was added to the brain to reach a protein concentration of approximately 200 mg/mL. The brain was sequentially homogenized using large and small clearance pestles (approximately ten strokes per pestle), contained in a tissue grinder set (2 mL, DWK Life Sciences Inc., Kimble Chase, Vineland, NJ, USA), and the homogenate was stored at − 80 °C until further use. The protein concentration was determined using a BCA kit (Micro BCA™ Protein Assay Kit, Thermo Fisher Scientific). Saturation assays were performed as described above. Homogenates were diluted in PBS to reach final protein concentrations of 0.5 mg/mL, 0.1 mg/mL, and 0.05 mg/mL, and fibrils were added to reach concentrations of 125 nM, 25 nM, and 5 nM.

### Radiosynthesis of [^11^C]MODAG-001 and (d_3_)-[^11^C]MODAG-001

Radiolabeling was performed in a manner analogous to [^11^C]anle253b radiolabeling [14]. For a detailed description, see supplementary material and methods section.

### Animals

All animal experiments were conducted in compliance with the European directives on the protection and use of laboratory animals (Council Directive 2010/63/UE) and in addition with the German animal protection law and with the approval of the local authorities (*Regierungspräsidium Tübingen*). Twenty-two female C57BL/6 mice (25 ± 6 g) and four female Sprague-Dawley rats (278 ± 25 g) were obtained from Charles River Laboratories (Sulzfeld, Germany). All animals were maintained in our vivarium on a 12:12 h light-dark cycle at a temperature of 22 °C with 40–60% humidity and given free access to a standard diet and tap water.

### In vivo PET imaging and metabolite analysis

PET imaging was performed on a dedicated small animal Inveon PET scanner (Siemens Healthcare, Knoxville (TN), USA). Mice and rats were anesthetized with 1.5–1.7% isoflurane evaporated in 100% oxygen at a flow rate of 0.8 L/min, which was maintained during the entire experiment. After a tail vein catheter was placed, the animal was positioned in the center of the field of view (FOV) on an MR-compatible PET bed that was connected to a feedback temperature control unit set to 37 °C. Mice were injected intravenously (i.v.) with 17 ± 2 MBq [^11^C]MODAG-001 (*n* = 3) or 12 ± 1 MBq (d_3_)-[^11^C]MODAG-001 (*n* = 5) 5 s after the start of the PET acquisition.

Rats were inoculated with αSYN fibrils (4 μL, 30 μM) into the right striatum (see supplementary material and methods section for a detailed description) and scanned 4 days post-inoculation with 23 ± 1 MBq of (d_3_)-[^11^C]MODAG-001.

Data acquisition, correction, framing and reconstruction, volume of interest (VOI) definition, and determination of binding parameters are described in detail in the supplementary material and methods section.

For metabolite analysis, mice were i.v. injected with 91 ± 24 MBq of [^11^C]MODAG-001 (*n* = 4 per time point) or 120 ± 41 MBq of (d_3_)-[^11^C]MODAG-001 (*n* = 3 per time point) using a tail vein catheter. Five and 15 min after injection, plasma and brain homogenates were analyzed using radio-HPLC (for detailed information see supplemental material and methods section).

### In vitro autoradiography in human brain tissue sections and continuous sucrose gradient centrifugation of human PD brain homogenates

Fresh-frozen 20 μm slices of the cortices from one AD case (Braak & Braak 5-6, CERAD C), two LBD cases (with pronounced αSYN and no to low Aβ and Tau pathology), one progressive supranuclear palsy (PSP) case, and one control case were kindly provided by Neurobiobank Munich [19], and stored at − 80 °C. PD brain tissue for preparation of homogenates was kindly provided by NBM [19]. Autoradiography, preparation of PD brain homogenates, and their analysis by continuous sucrose gradient centrifugation experiments are described in the supplementary material and methods section.

## Results

### Fibril characterization and in vitro binding assays

**Fig. 2:**
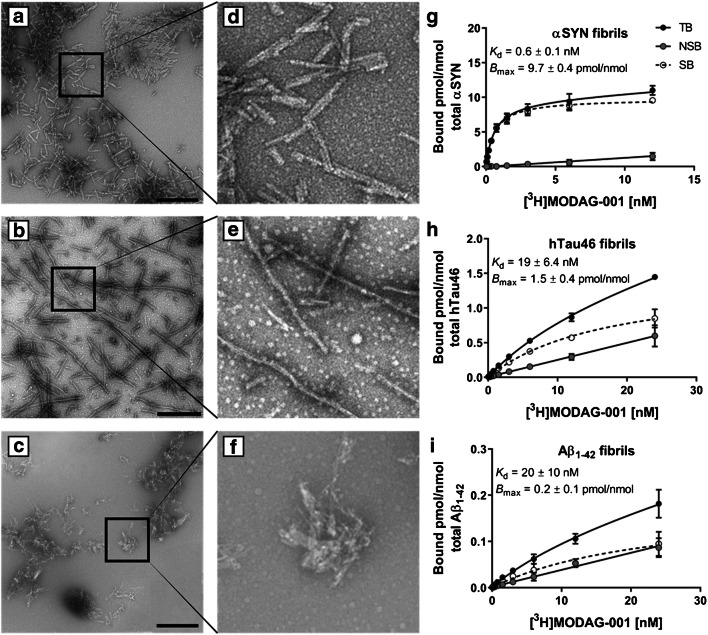
[^3^H]MODAG-001 binding experiments using recombinant human fibrils. Negative stain electron microscopy images and 4.7-fold magnification of α-synuclein (**a**, **d**), hTau46 (**b**, **e**), and amyloid-β_1–42_ (**c**, **f**) fibrils used in the binding experiments. Total and nonspecific binding curves of [^3^H]MODAG-001 to α-synuclein (**g**), hTau46 (**h**), and amyloid-β_1–42_ (**i**) fibrils. Scale bar 500 nm. TB, total binding; NSB, nonspecific binding; SB, specific binding; αSYN, α-synuclein; Aβ_1–42_, amyloid-β_1–42_

Quality control of in vitro-formed fibrils using negative stain electron microscopy (EM) (Fig. 2a–f and Supplemental Fig. S2) confirmed the presence of typical amyloid fibrils. In saturation binding assays, [^3^H]MODAG-001 showed a very high affinity towards αSYN fibrils (*K*_d_ = 0.6 ± 0.1 nM) with low nonspecific binding (Fig. 2g). Compared to the affinity towards αSYN fibrils, a 30-fold lower affinity was observed towards hTau46 fibrils (*K*_d_ = 19 ± 6.4 nM) (Fig. 2h) and Aβ_1–42_ fibrils (*K*_d_ = 20 ± 10 nM) (Fig. 2i). Of note, also *B*_max_ was almost 7 and 50-fold higher for αSYN fibrils than for hTau46 fibrils and Aβ_1–42_ fibrils, respectively.

To further characterize the potential binding site of [^3^H]MODAG-001, a competition assay with SIL26 was performed. SIL26 displaced [^3^H]MODAG-001 binding in a dose-dependent manner with a *K*_i_ of 21 nM, indicating lower affinity for SIL26 than for MODAG-001 (see Supplemental Fig. S3).

### Determination of the limit of detection in brain homogenates

**Fig. 3:**
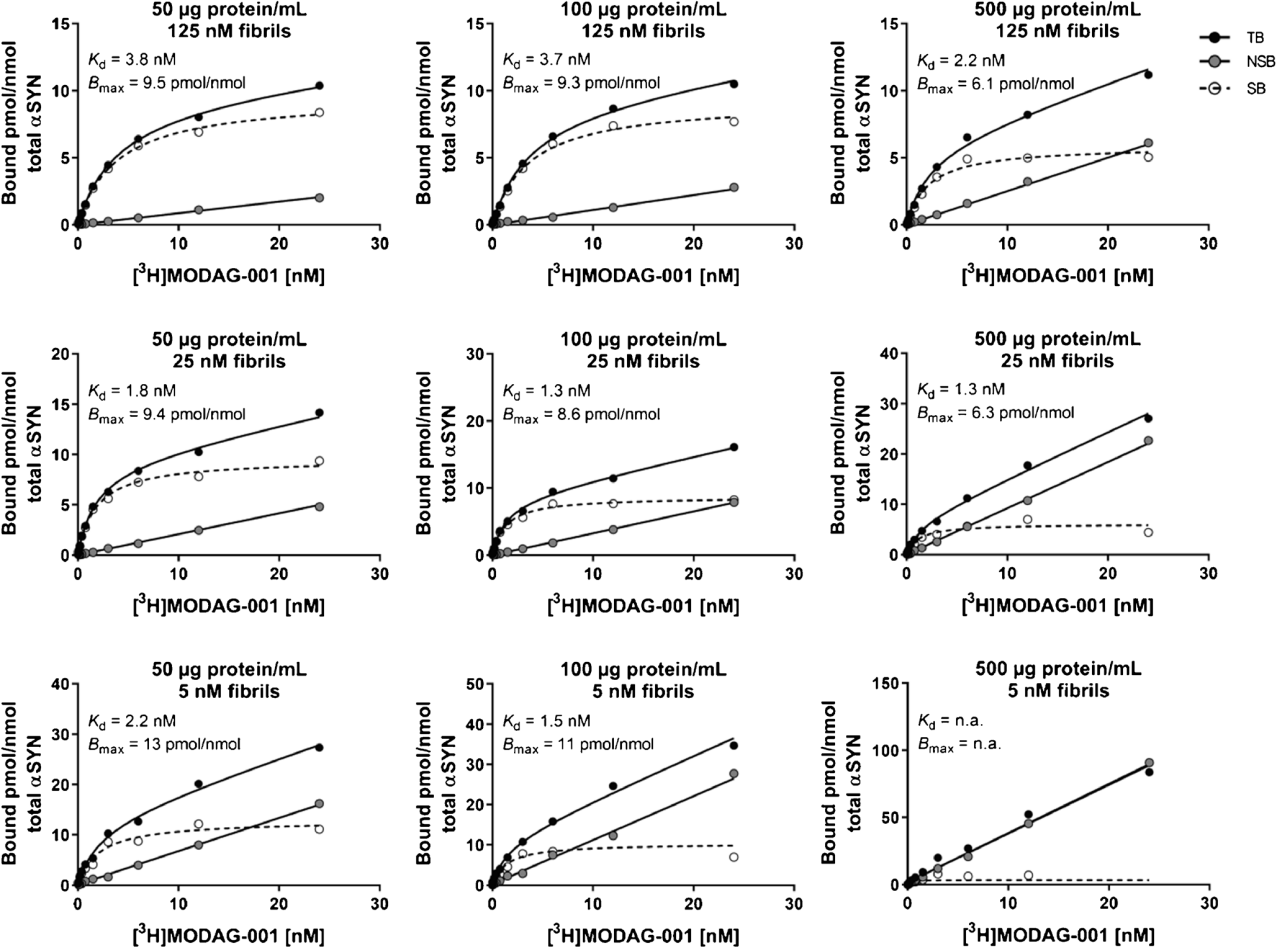
[^3^H]MODAG-001 sensitivity analysis. Determination of total binding and nonspecific binding of [^3^H]MODAG-001 in mouse brain homogenates spiked with recombinant α-synuclein (αSYN) fibrils. Increasing homogenate protein concentrations (left to right) were inoculated with decreasing fibril concentrations (top to bottom). At smallest concentrations of 5 nM fibrils, total binding and nonspecific curves were still separated to calculate specific binding at a homogenate concentration of 100 μg/mL, but indistinguishable at a protein concentration of 500 μg/mL. A 5-fold increase of the αSYN fibril concentration increased the total binding curve, enabling the calculation of specific binding. TB, total binding; NSB, nonspecific binding; SB, specific binding; αSYN, α-synuclein; Aβ_1–42_, amyloid-β_1–42_

To determine the limit of detection of αSYN fibrils by [^3^H]MODAG-001 in brain tissue homogenates, a saturation binding assay was performed in mouse brain homogenates spiked with αSYN fibrils at different concentrations (Fig. 3). Separation of the total binding (TB) and nonspecific binding (NSB) curves to calculate specific binding (SB) in the presence of 100 μg protein/mL mouse brain homogenate was possible at αSYN concentrations down to 5 nM (limit of detection). At a 5-fold higher homogenate concentration of 500 μg protein/mL, SB was undetectable, as the SB curve was superposed by the NSB curve, but SB was detectable when the fibril concentration was increased to 25 nM.

Using sucrose gradient centrifugation to determine the amount of aggregated αSYN in a human PD brain homogenate, we observed an αSYN aggregate concentration of approximately 4 nM and 830 μg protein/mL in a 1% homogenate, which is slightly below the limit of detection by [^3^H]MODAG-001 (Supplemental Fig. S5).

### ^11^C-labeling of MODAG-001 and optimization of the molar activity for in vivo applications

**Fig. 4:**
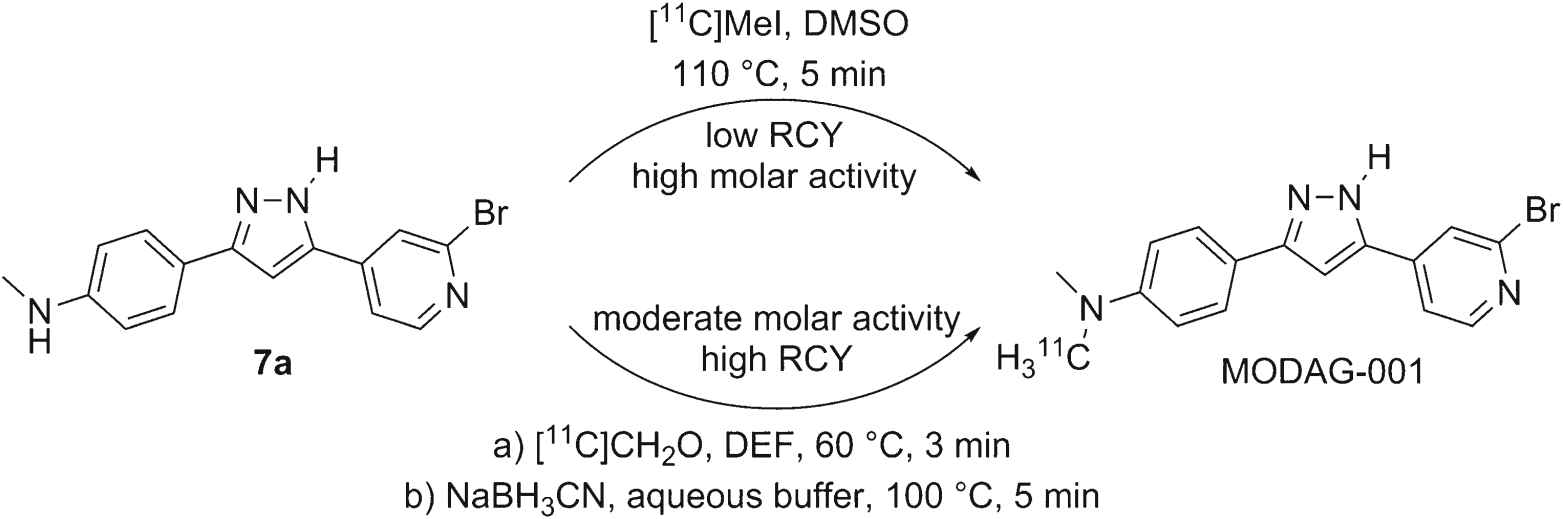
Radiolabeling of [^11^C]MODAG-001. Depending on the required molar radioactivity, two different strategies were used. Direct methylation (upper reaction conditions) gave lower radiochemical yields but a high molar radioactivity (266 ± 113 MBq, 98.6 ± 24.7 GBq/μmol) while reductive methylation (lower reaction arrow) gave better yields but lower molar radioactivities (1003 ± 247 MBq, 31.3 ± 6.4 GBq/μmol)

MODAG-001 was readily radiolabeled by reductive methylation using in situ-generated formaldehyde with a radiochemical yield of 11.4 ± 3.7% (*n* = 18, decay-corrected from [^11^C]MeI) (Fig. 4). These preparations were suitable for the first in vivo studies (similar to anle253b [14]), but the A_m_ was low (31.3 ± 6.4 GBq/μmol), prompting us to switch to direct methylation for subsequent experiments. Here, we achieved a A_m_ of 98.6 ± 24.7 GBq/μmol but a radiochemical yield of only 3.6 ± 1.1% (final product: 266 ± 113 MBq instead of 1003 ± 247 MBq), a yield sufficient for our animal studies.

### Pharmacokinetic and metabolic profile of [^11^C]MODAG-001 in mice

**Fig. 5:**
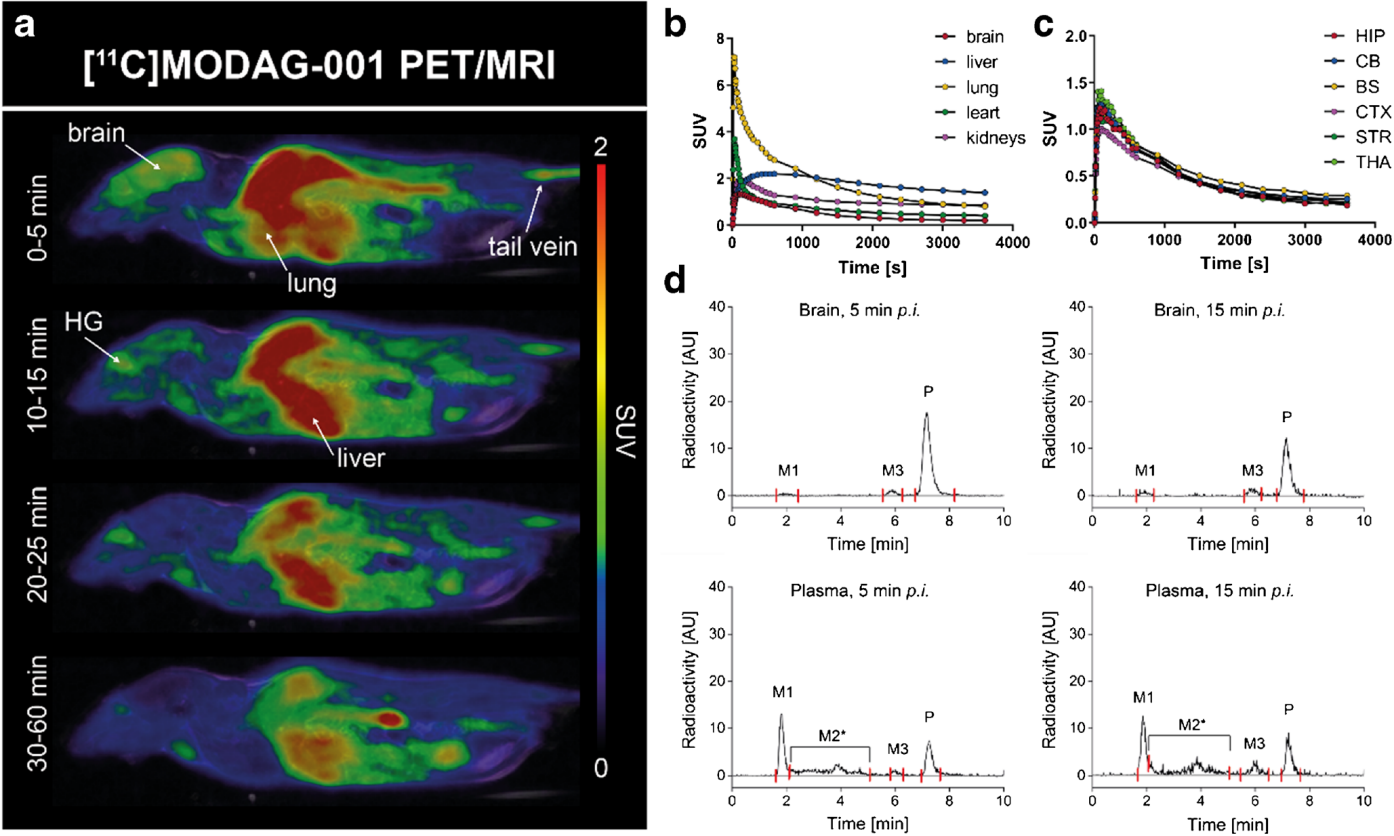
Pharmacokinetic profile of [^11^C]MODAG-001 in the mouse. Whole-body PET/MR images of one exemplary mouse at different time points after i.v. injection (**a**) and corresponding time activity curves of different organs and brain regions (**b**, **c**). Images show a good brain uptake with a peak standardized uptake value of 1.4. Metabolite analysis of [^11^C]MODAG-001 revealed two detectable metabolites M1 and M3 present in the brain (**d**). PET, positron emission tomography; MRI, magnetic resonance imaging; SUV, standardized uptake value; HG, harderian glands; HIP, hippocampus; CB, cerebellum; BS, brainstem; CTX, cortex; STR, striatum; THA, thalamus; AU, arbitrary units; M1, metabolite 1; M2*, mixture of various metabolites; M3, metabolite 3; P, parent compound; *p.i.*, *post iniectio*

Figure 5 shows whole-body sagittal [^11^C]MODAG-001 PET/MR images and time activity curves (TACs) of selected organs from one exemplary animal at different time points after i.v. injection, which resulted in rapid brain uptake with an SUV of 1.4 (radioactivity normalized to injected dose and body weight) with only small regional differences across multiple brain regions and fast washout from the brain (Fig. 5a–c).

Radio-metabolite formation of [^11^C]MODAG-001 in the mouse brain and plasma was determined 5 min and 15 min after tracer injection (Fig. 5d). A full quantitative analysis of two exemplary mice revealed two metabolites in the mouse brain and three metabolites in the plasma with 91% and 81% of the parent compound present in the brain at 5 min and 15 min after injection, respectively. Quantitative values determined by brain and plasma analyses are summarized in Table 1.

**Table 1:** Quantification of radio-metabolites in plasma and brain samples 5 and 15 min after [^11^C]MODAG-001 injection

	M1		M2*		M3		P	
	**%**	%ID	%	%ID	%	%ID	%	%ID
Brain, 5 min	3	0.4*	–	–	6	0.7*	91	10.5*
Brain, 15 min	7	0.4*	–	–	12	0.7*	81	5.3*
Plasma, 5 min	34	1.0^#^	37	1.1^#^	3	0.1^#^	26	0.8^#^
Plasma, 15 min	29	0.8^#^	36	1.0^#^	11	0.3^#^	24	0.7^#^

*M1*, metabolite 1; M2*, mixture of various metabolites; *M3*, metabolite 3; *P*, parent compound; *%ID*, % injected dose; * per g brain tissue, # per plasma in 1 mL blood

As we hypothesize that the NMe_2_ group is the major target of metabolism for [^11^C]MODAG-001, we deuterated [^11^C]MODAG-001, as previous studies have shown the utility of this method by enhancing the in vivo stability of compounds and drugs injected into animals as well as humans [22]. Therefore, a deuterium-substituted analog of [^11^C]MODAG-001 was designed by full deuteration of the nonradioactive methyl group (Fig. 1d).

### Pharmacokinetic and metabolic profile of (d_3_)-[^11^C]MODAG-001 in mice

**Fig. 6:**
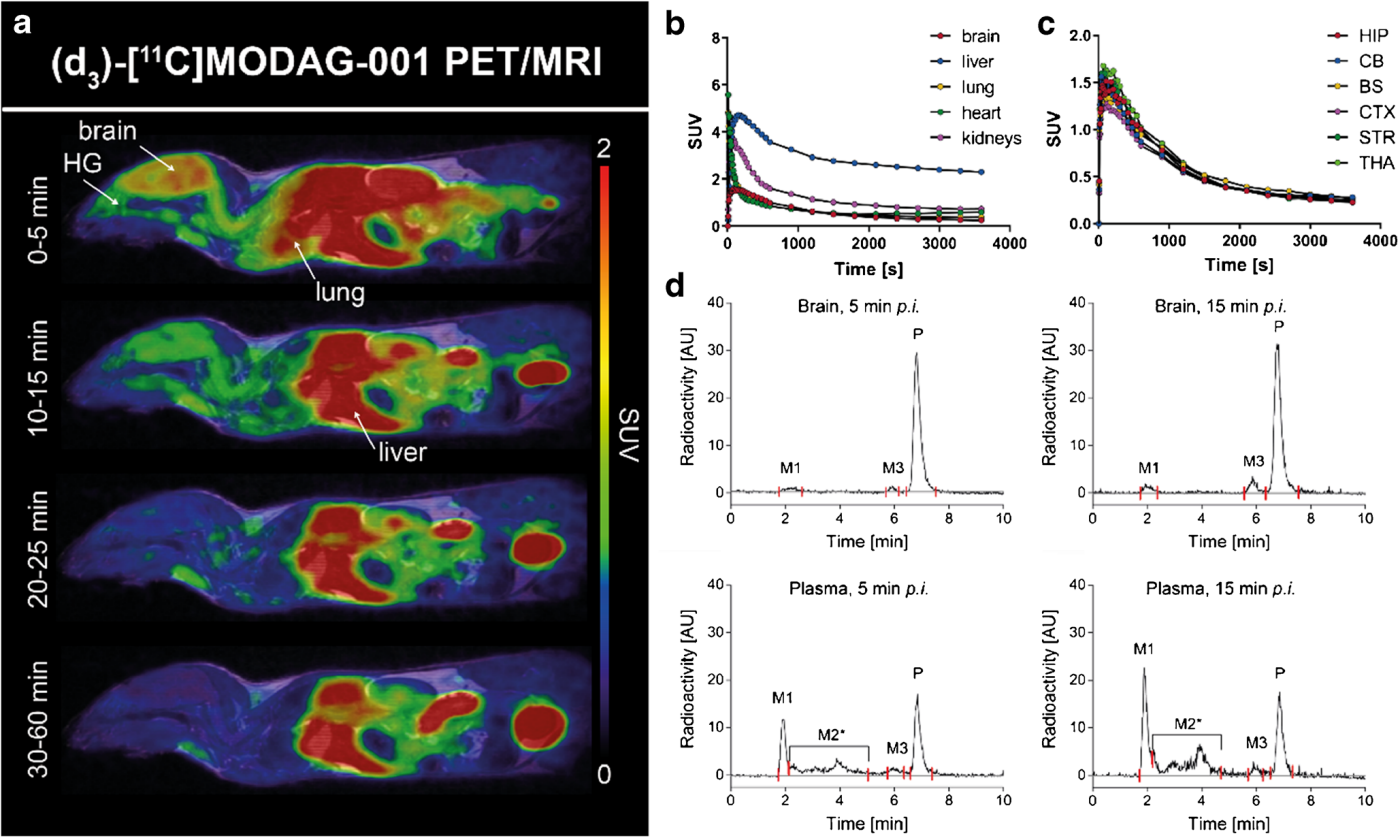
Pharmacokinetic profile of (d_3_)-[^11^C]MODAG-001 in the mouse. Whole-body tracer accumulation over time is shown (**a**, **b**). (d_3_)-[^11^C]MODAG-001 rapidly entered the brain with peak standardized uptake values of 1.7 (**c**) followed by a fast washout. HPLC chromatograms of (d_3_)-[^11^C]MODAG-001 in plasma and brain homogenates revealed two detectable metabolites M1 and M3 present in the brain (**d**). HPLC, high-performance liquid chromatography, PET, positron emission tomography; MRI, magnetic resonance imaging; SUV, standardized uptake value; HG, harderian glands; HIP, hippocampus; CB, cerebellum; BS, brain stem; CTX, cortex; STR, striatum; THA, thalamus; AU, arbitrary units; M1, metabolite 1; M2*, mixture of various metabolites; M3, metabolite 3; P, parent compound; *p.i.*, *post iniectio*

After the successful synthesis of (d_3_)-[^11^C]MODAG-001, dynamic whole-body PET scans were performed in mice. Figure 6 a–c shows sagittal whole-body PET/MR images and TACs of selected organs from one exemplary mouse at different time points after systemic administration of (d_3_)-[^11^C]MODAG-001, which resulted in rapid brain uptake with an SUV of 1.7 (radioactivity normalized to injected dose and body weight) with only small regional differences across multiple brain regions.

Chromatograms showed similar results of all metabolite experiments (Fig. 6d). Quantitative analysis of two exemplary mice revealed two radio-metabolites in the brain and three radio-metabolites in the plasma at 5 and 15 min after tracer injection, with 93% and 87% of the parent compound present in the brain at 5 and 15 min after injection, respectively. Quantitative values determined by brain and plasma analyses are summarized in Table 2.

**Table 2:** Quantification of radio-metabolites in plasma and brain samples 5 and 15 min after (d_3_)-[^11^C]MODAG-001 injection

	M1		M2*		M3		P	
	%	%ID	%	%ID	%	%ID	%	%ID
Brain, 5 min	5	0.4*	–	–	2	0.2*	93	8.8*
Brain, 15 min	5	0.4*	–	–	8	0.7*	87	7.2*
Plasma, 5 min	21	0.6^#^	34	0.9^#^	6	0.1^#^	39	1.0^#^
Plasma, 15 min	30	0.8^#^	36	1.0^#^	4	0.1^#^	30	0.8^#^

*M1*, metabolite 1; M2*, mixture of various metabolites; *M3*, metabolite 3; *P*, parent compound; *%ID*, % injected dose; * per g brain tissue, # per plasma in 1 mL blood

### Binding of (d_3_)-[^11^C]MODAG-001 to αSYN in a fibril-inoculated rat brain

**Fig. 7:**
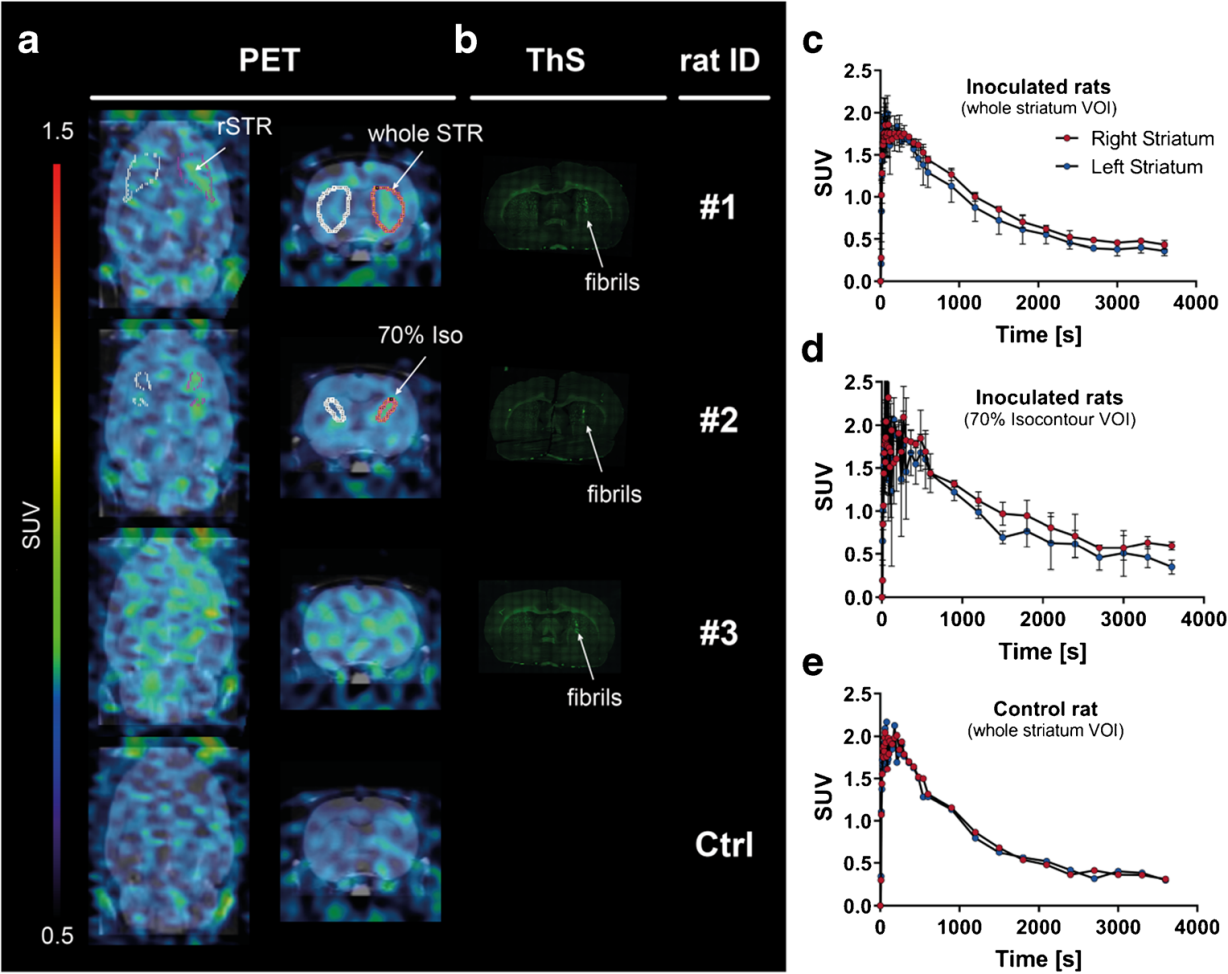
In vivo binding of (d_3_)-[^11^C]MODAG-001 in α-synuclein-inoculated rats. Coronal and transversal PET images summed up from 2.5 to 60 min of three rats (#1–3) 4 days *post inoculation* and one non-injected control rat (**a**). Images show increased tracer accumulation in the αSYN fibril-inoculated right striatum compared to the vehicle-injected contralateral striatum (**a**, row 1–3). Thioflavin S staining (**b**) confirmed the location of αSYN fibrils (white arrow) in the right striatum of fibril-inoculated rats (#1–3) (**b**). Time activity curves of (d_3_)-[^11^C]MODAG-001 show a rapid brain uptake with peak standardized uptake values of 2.1 ± 0.1 in the left striatum and a difference between the right (injected) and left (vehicle injected) striatum (**c**, **d**, **e**). The binding potential (DVR-1_40-60min_) was 0.14 ± 0.1 for the whole striatum VOI analysis and 0.44 ± 0.21 for the 70% isocontour VOI analysis, using the contralateral side as reference region (**c**), while no difference was observed in the non-inoculated control rat (**d**). PET, positron emission tomography; αSYN, α-synuclein; rSTR, right striatum; ThS, thioflavin S; Ctrl, control; SUV, standardized uptake value, DVR-1, distribution volume ratio-1; VOI, voxel of interest

Figure 7 a shows coregistered PET/MR images of three fibril-inoculated rats and one non-injected rat. Thioflavin S staining confirmed the presence of fibrils at the site of inoculation in the right striatum (Fig. 7b). Using a SUV threshold of 0.8 to 1.2 to remove background binding, a better visualization of the tracer binding to the inoculated fibrils was possible from the images (see Supplemental Fig. S4). (d_3_)-[^11^C]MODAG-001 injection resulted in rapid brain uptake, with peak SUV values of 2.1 ± 0.1 in the left striatum of inoculated rats (radioactivity normalized to injected dose and body weight) and 2.1 in the non-injected rat, and fast washout from the brain (Fig. 7c–e) with higher tracer retention in the right, fibril-inoculated striatum compared to the contralateral, vehicle-injected striatum. Using a conservative analytical approach with the whole striatum as the reference region, a mean distribution volume ratio (DVR)-1_40-60min_ of 0.14 ± 0.1 was calculated, while a mean DVR-1_40-60min_ of 0.44 ± 0.21 was calculated using 70% automatic isocontour detection at the location of fibril accumulation. The mean SUV_40-60min_ of the right, fibril-inoculated striatum and contralateral, vehicle-injected striatum significantly differed (*p* = 0.03 with the whole striatum as the VOI). The non-injected rat showed no difference in mean SUV_40-60min_ between the right and left striatum, with brain uptake comparable to that in the inoculated rats. In all rat brains, nonspecific binding was also observed in brain regions without fibrils, reducing the SNR in the inoculated striatum.

### In vitro binding of [^3^H]MODAG-001 to human brain tissue

**Fig. 8:**
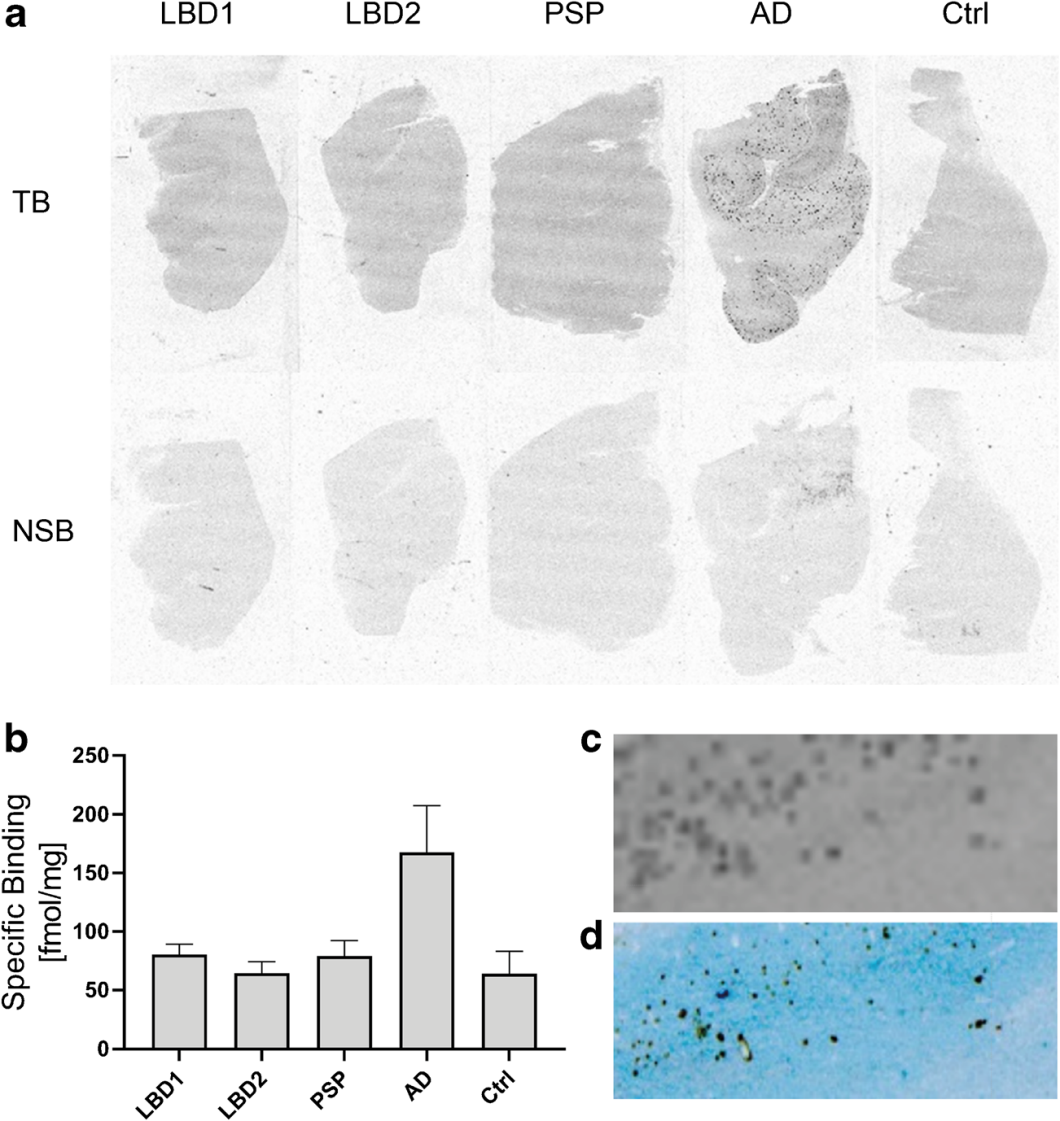
**a** [^3^H]MODAG-001 in vitro autoradiography (AR) on human brain slices with different pathologies: Lewy body dementia (LBD), progressive supranuclear palsy (PSP), Alzheimer’s disease (AD), healthy control (Ctrl). Images show total (TB) and nonspecific binding (NSB) of [^3^H]MODAG-001. **b** Quantification of specific tracer binding in respective brain slices in fmol/mg is shown. **c** Magnification of a pathological region in the AR of an AD case counterstained for Aβ plaques (**d**)

Figure 8 a shows [^3^H]MODAG-001 AR images of the cortex of two LBD cases, one AD case and one PSP case, and one control case. In all cases, [^3^H]MODAG-001 showed a nonspecific blocking effect, which was likely related to the high concentration of MODAG-001 (50 μM) used for blocking. For the AD case, clear binding of [^3^H]MODAG-001 in the cortex was observed. Binding values for the LBD 1 (80.5 ± 8.7 pmol/mg), LBD 2 (64.4 ± 9.8 pmol/mg), PSP (79.0 ± 13.3 pmol/mg), and AD (167.7 ± 40.0 pmol/mg) cases and the control (64.1 ± 19.1 pmol/mg) are shown in Fig. 8b. At high magnification, labeling of plaque-like structures was evident in the AD case (Fig. 8c). Comparison with the results of immunohistochemical staining in consecutive sections confirmed that this signal colocalized with Aβ-positive AD plaques (Fig. 8d).

## Discussion

Since the discovery in 1997 that the protein αSYN can be found in Lewy bodies in PD [1], much research on αSYN has been conducted. The aggregation of αSYN seems to be the central driver of the pathogenesis of synucleinopathies; however, the relationship between αSYN misfolding and cellular dysfunction or cell death is far from fully understood. The removal of αSYN aggregates holds considerable promise as a therapeutic strategy. In this context, imaging biomarkers are desperately needed for early and differential diagnosis, to follow disease progression and to determine the efficacy of potential disease-modifying treatments. An αSYN PET tracer would unquestionably be of high importance and a game-changing tool for diagnosis and therapy development.

Here, we aimed to develop a PET tracer based on the lead structure of anle138b by further modifying anle253b, which was previously reported by our group to have a good affinity towards human recombinant αSYN fibrils but poor pharmacokinetics in vivo, presumably caused by high lipophilicity with a calculated log*P* (clog*P*) of 5.21 [14]. Ishikawa et al. performed studies on the improvement in polarity of drug candidates. They showed that exchange of the phenyl group with pyridine largely improved polarity in phosphate buffer at physiological pH, which consequently reduced the log*P* value [23]. We designed a chemical derivative of anle253b, MODAG-001, in which one of the phenyl groups was exchanged with pyridine to reduce the lipophilicity of the compound to a clog*P* value of 3.85. After tritiation, [^3^H]MODAG-001 was tested in saturation binding experiments against human recombinant fibrils and showed very high affinity to αSYN fibrils, with a *K*_d_ value of 0.6 nM in vitro. Compared to [^3^H]MODAG-001, all the compounds, which have been described in the literature as potential αSYN PET ligands, showed an at least one order of magnitude lower binding affinity (higher *K*_d_ values) in vitro towards recombinant αSYN fibrils [14, 21, 24–31]. These compounds comprise [^125^I]SIL23, (*K*_d_ = 148 nM) and its derivative SIL26 (*K*_i_ = 15.5 nM), [24] [^18^F]BF-227 (*K*_d_ = 9.6 nM) [26], several fluorescent probes (*K*_d_s in the micromolar and elevated nanomolar range) [32, 33], and the ^18^F-labeled 3-(benzylidine)indolin-2-one derivative [^18^F]46a (*K*_d_ = 8.9 nM) [25].

Furthermore, we observed 30-fold selectivity over hTau46 and Aβ_1–42_ fibrils, which was suggested for an ideal CNS PET tracer, as both structurally similar proteins have also been shown to be highly abundant in many patients with synucleinopathies [34]. However, the selectivity is determined not only by a lower *K*_d_, but also by the difference of available binding sites. *B*_max_ was approximately 7- and 50-fold higher for αSYN fibrils compared to hTau46 fibrils and Aβ_1–42_ fibrils, respectively. The measured fibril length of hTau46 (453.4 ± 261.9 nm) was three times larger compared to αSYN (152.5 ± 76.6 nm) and Aβ_1–42_ (139.4 ± 77.3 nm), which were very similar in size. Of note, not only the fibril length, but especially the amount of available binding sites per fibril length, which might differ due to differences in the 3D structure, may play an important role.

These encouraging in vitro binding data were the prerequisite for further ^11^C-labeling and in vitro and in vivo characterization of MODAG-001. The pharmacokinetic and metabolic profiles of [^11^C]MODAG-001 after i.v. injection into healthy mice revealed good BBB penetration with high uptake into the brain (SUV = 1.4) and relatively fast clearance from the brain. Our metabolite analysis revealed that [^11^C]MODAG-001 was degraded into the three metabolites, two of which were detectable in the brain, with 81% of the parent compound remaining at 15 min. We hypothesize that metabolite M3 might be the demethylated form of MODAG-001, as demethylation is a very common form of metabolic degradation. Fast metabolism resulting in BBB-penetrating radio-metabolites hampers tracer quantification. Previous studies have shown that improved metabolic stability can be obtained by incorporating deuterium into the molecule. In comparison to carbon-hydrogen bonds, carbon-deuterium bonds potentially decelerate metabolism by cytochrome P450 enzymes due to the primary kinetic isotopic effect [22]. Since the NMe_2_ group of MODAG-001 is considered the main target of metabolism (M3 corresponds to the monodemethylated metabolite of MODAG-001), we fully deuterated the nonradioactive methyl group to reduce the formation of radio-metabolites and improve the metabolic stability of the tracer in vivo. Comparison of the pharmacokinetic and metabolic profiles of (d_3_)-[^11^C]MODAG-001 and [^11^C]MODAG-001 revealed that formation of the monodemethylated radio-metabolite M3 was reduced and that the ratio of M3 to M1 (which hypothetically represents the cleaved ^11^C-methyl group) was increased for (d_3_)-[^11^C]MODAG-001. Moreover, total metabolism of the parent compound was reduced, resulting in increased levels of the parent compound in the brain at 15 min. This is emphasized in Table 1 and Table 2 showing that the injected dose per gram for [^11^C]MODAG-001 was reduced from 10.5% at 5 min to 5.3% at 15 min, whereas the injected dose per gram for (d_3_)-[^11^C]MODAG-001 was reduced to a lower extent from 8.8% at 5 min to 7.2% at 15 min.

We next asked whether (d_3_)-[^11^C]MODAG-001 can detect aggregated αSYN in the brain and thus inoculated the right striatum in rats with the same batch of αSYN fibrils used for the in vitro binding experiments. (d_3_)-[^11^C]MODAG-001 binding properties in three fibril-inoculated rats and one non-injected control rat were assessed using in vivo PET imaging. In the three fibril-inoculated rats, we observed higher binding in the right striatum compared to the vehicle-inoculated contralateral side, with mean DVR-1_40-60min_ of 0.14 ± 0.1 (whole striatum) and 0.44 ± 0.21 (70% automatic isocontour detection) 4 days after injection, respectively, indicating in vivo binding of (d_3_)-[^11^C]MODAG-001 to inoculated αSYN fibrils. Furthermore, similar overall brain uptake was observed in fibril-inoculated rats (peak SUV, 2.1 ± 0.1) and the non-injected rat (peak SUV, 2.1), demonstrating that potential disruption of the BBB from the intracranial injection is unlikely to account for higher uptake into the inoculated striatum. A similar rat model was also used by Verdurand et al. [30] to select potential αSYN PET tracer candidates. Notably, they were able to detect the binding of [^18^F]BF227, [^18^F]2FBox, and [^18^F]4FBox to αSYN and Aβ_1–42_ fibrils using in vitro AR; however, no binding was observed in vivo despite good uptake into the cerebral tissue and high A_m_ (68–543 GBq/μmol).

To further test binding of MODAG-001 in human brain slices with confirmed αSYN pathology, we performed in vitro AR using [^3^H]MODAG-001 in LBD, PSP, AD, and control cases [19]. The advantage of using tritium AR over carbon-11 AR is its 10-fold higher spatial resolution of approximately 50 μm^2^ compared to the spatial resolution of carbon-11 AR of approximately 566 μm^2^ due to the positron range of the radionuclide [35, 36].

Despite the high affinity of [^3^H]MODAG-001 for recombinant αSYN fibrils and good selectivity for αSYN fibrils over hTau46 and Aβ_1–42_ fibrils, no strong binding was observed in brain sections from LBD cases. We observed slightly more intense binding in the cortical gray matter compared to the white matter, which could be blocked by the nonlabeled compound, but this signal was not significantly stronger than that in controls. Interestingly, clear, blockable binding corresponding to the distribution of Aβ plaques was observed in AD brain tissue. Whether this represents binding to Aβ fibrils or binding to aggregated αSYN species, which have been described in AD plaques, remains to be determined [37–40]. Notably, no evidence of binding to aggregated Tau was observed in the PSP and AD cases. A high SNR is an important requirement in brain binding studies with low target abundancy. Possible explanations why we did not observe stronger binding in LBD brain sections are likely related to low target availability in the brains of LBD patients, high nonspecific binding, and/or structural differences between the αSYN fibrils used in the screening assays and those in human brain tissue.

We determined the limit of detection of [^3^H]MODAG-001 using fibrils and brain homogenate at different concentrations. Quantification of specific binding was still possible at αSYN concentrations down to 5 nM in the presence of 100 μg protein/mL mouse brain homogenate, but not at higher homogenate concentrations of 500 μg protein/mL. When we used sucrose gradient centrifugation of human PD brain tissue to quantify aggregated αSYN, we found approximately 400 nM aggregated αSYN, corresponding to 4 nM aggregated αSYN and 830 μg protein/mL in homogenate at a 1:100 dilution (Supplemental Fig. S5c). At this fibril concentration, the homogenate concentration was approximately 8-fold higher than the limit for a similar fibril concentration determined in our assay. Therefore, we hypothesize that the nonspecific binding of [^3^H]MODAG-001 is likely responsible for the low signal-to-noise ratio in pathological human brain tissue. For MODAG-001, we calculated a clog*P* value of 3.85, which is rather high compared to the values calculated for successfully established PET ligands.

Structural differences between recombinant fibrils obtained in vitro and fibrils in the LB and LN of LBD patients may also account for the different binding behaviors of MODAG-001. Such structural differences were identified in a study in which αSYN fibrils from LBD patients were amplified from in vivo aggregates by protein misfolding cyclic amplification (PMCA) [41]. The same study also revealed the heterogeneity of αSYN fibrils in different synucleinopathies using solution-state NMR spectroscopy and fluorescent probes. Furthermore, structural differences were also observed in cryo-EM studies in which artificially produced Tau fibrils were compared with Tau extracted from AD or Pick’s disease patients with Tau pathology [42–45].

Despite the presence of structural differences between recombinant fibrils and those found in human brain slices, homogenates, or αSYN brain extracts, the availability of the latter is very limited; as such, recombinant fibrils remain a widely used screening tool for the preselection of compounds before PET radiolabeling.

Many studies have shown that a large percentage of AD patients exhibit significant LB pathology in addition to Aβ plaques and neurofibrillary tangles (NFTs) and vice versa [46–50]. As an example, Hamilton et al. [47] investigated the *postmortem* tissue of 145 sporadic AD cases using immunohistochemistry for αSYN and observed LBs in 60.7% of all cases. Colo-Cadena et al. [48] found that Aβ deposition positively correlated with LBD pathogenesis. However, direct comparison of the results of AR and immunohistochemistry in the same AD brain slice revealed the clear colocalization of Aβ-positive plaques and bound MODAG-001. Therefore, the binding is either related to cross-β-sheet structures, a common feature shared by Aβ, Tau, and αSYN [51–53], or to the non-amyloid-β component (NAC) domain identified by Ueda et al., which is part of the αSYN protein in AD plaques [40]. However, as we cannot provide experimental evidence for the latter, this remains speculative.

Determination of the αSYN fibril-binding sites of novel compounds is crucial for PET tracer development. Hsieh and Ferrie et al. identified three putative binding sites for fibrillar αSYN using a combination of in silico docking, photoaffinity labeling, and radiotracer binding studies [54]. While styrene- and piperazine-based analogs showed a preference for sites 2 and 9, tricyclic compounds and an indolinone-diene analog showed a preference for site 3/13. We performed a [^3^H]MODAG-001 competition assay using the tricyclic compound SIL26 as a competitor and observed a *K*_i_ value of 21 nM, indicating preferential binding to site 3/13 at fibrillar αSYN. However, the interpretation needs to be done with care, as preference to sites 2 and 9 were not tested.

## Conclusion

Over the past years, several attempts have been made to develop an αSYN-specific PET tracer [14, 21, 24–31]. However, the low abundance of αSYN inclusions and high similarity to structurally related proteins present in pathological brain regions [51, 55] made its discovery very challenging. MODAG-001 fulfills several important criteria needed for a CNS PET tracer targeting αSYN. Its nonspecific binding needs to be reduced to achieve a higher SNR in human brain tissue with synuclein pathology, which is currently being addressed in further optimization experiments. To the best of our knowledge, none of the compounds available so far has been shown to have a comparably high affinity towards recombinant αSYN fibrils in the picomolar range. In addition, MODAG-001 showed high brain uptake and favorable in vivo kinetics and biodistribution in rats and mice.

## Supplementary Information

ESM 1 (DOCX 948 kb)

## Data Availability

The data can be made available upon request.
