# Supplementary material for: [11C]MODAG-001—towards a PET tracer targeting α-synuclein aggregates
Source: Eur J Nucl Med Mol Imaging. 2020 Dec 28;48(6):1759–72. doi: 10.1007/s00259-020-05133-x (PMC8113290; doi:10.1007/s00259-020-05133-x)
Supplement: Supplementary file 1 — (DOCX 948 kb) [file 259_2020_5133_MOESM1_ESM.docx]

**Supplemental Material:**

**[^11^C]MODAG‑001 – Towards a PET Tracer Targeting α‑Synuclein Aggregates**

Laura Kuebler^1*^, Sabrina Buss^1*^, Andrei Leonov^2,3*^, Sergey Ryazanov^3*†^, Felix Schmidt^2*^, Andreas Maurer^1^, Daniel Weckbecker^2^, Anne M Landau^4,5^, Thea P Lillethorup^5^, Daniel Bleher^1^, Ran Sing Saw^1^, Bernd J Pichler^1^, Christian Griesinger^3,6#^, Armin Giese^2#^, Kristina Herfert^1#^

^*^contributed equally

^#^corresponding authors

^†^present address also MODAG

^1^Werner Siemens Imaging Center, Department of Preclinical Imaging and Radiopharmacy, Eberhard Karls University, Tuebingen, Germany

^2^MODAG GmbH, Wendelsheim, Germany

^3^Department of NMR-based Structural Biology, Max Planck Institute for Biophysical Chemistry, Göttingen, Germany

^4^Translational Neuropsychiatry Unit, Aarhus University, Aarhus, Denmark

^5^Department of Nuclear Medicine and PET, Aarhus University, Aarhus, Denmark

^6^University Göttingen, Cluster of Excellence Multiscale Bioimaging molecular machines, 37077 Göttingen, Germany

**Material and methods**

**Compound Synthesis**

All starting materials and solvents were of commercial grade and used as received unless noted otherwise. Thin layer chromatography (TLC): Macherey‑Nagel precoated sheets, 0.25 mm ALUGRAM® SIL G/UV254 plates, detection with UV and/or by charring with 10 wt % ethanolic phosphomolybdic acid reagent followed by heating at 200 °C. Flash column chromatography was performed by using Merck silica gel 60 (0.063‑0.100 mm). Analytical high performance liquid chromatography (HPLC) was performed by using a Waters HPLC system with a Waters 996 Photodiode Array Detector. All separations involved a mobile phase of 0.1 % trifluoroacetic acid (TFA) (v/v) in water and 0.1 % TFA in acetonitrile. Unless otherwise specified, a gradient 50 % CH_3_CN /50 % H_2_O →100 % CH_3_CN in 30 minutes was used. HPLC was performed by using a reversed-phase (RP) column Eurospher RP 18, 100 Å, 5 μm, 250 × 4.6 mm at flow rate of 1 mL/min. Electrospray ionization mass spectrometry (ESI-MS) and liquid chromatography/ mass spectrometry (LC/MS) analyses were obtained by using a Waters Micromass ZQ 4000 mass spectrometer in conjunction with the Waters HPLC apparatus described above. NMR spectra were recorded by using a 400 MHz Bruker Avance spectrometer (Bruker AG, Rheinstetten, Germany) equipped with a TXI HCN z-gradient probe. All spectra were processed by using TOPSPIN 3.1 (Bruker AG, Karlsruhe, Germany). ^1^H-NMR chemical shifts (δ) are reported in parts per million (ppm) relative to CHCl_3_, DMSO-d_5_ and TFA-d_1_ as internal standards. Data are reported as follows: chemical shift, multiplicity (s = singlet, d = doublet, t = triplet, q = quartet, qi = quintet, dd = doublet of doublets, dt = doublet of triplets, b = broadened, m = multiplet, sept = septet), coupling constants (J, given in Hz), integration. ^13^C-NMR chemical shifts (δ) are reported in parts per million (ppm) relative to CDCl_3_, DMSO-d_6_ and TFA-d_1_ as internal standards. The following experiments were used to record the resonances of the compounds: ^1^H-1D, ^13^C-1D-NMR spectra and ^13^C-APT (attached proton test with a single J-evolution time of 1/145 seconds, spectra are processed such that quaternary and methylene groups have positive sign and methyl and methine groups negative sign). To resolve overlap of resonances and recover undetectable resonances in ^1^H and APT spectra, 2D-[^13^C,^1^H]-HSQC (heteronuclear single quantum coherence), 2D-[^13^C,^1^H]-HMBC (heteronuclear multiple bond correlation) and 2D-NOESY were recorded for some compounds.

## Experimental procedures and characterization of compounds

**MODAG‑001:** 4-[3-(4-Dimethylaminophenyl)-1*H*-pyrazol-5-yl]-2-bromopyridine

To a solution of 1-[4-(dimethylamino)phenyl]ethanone (490 mg, 3.00 mmol) and methyl 2-bromopyridine-4-carboxylate (843 mg, 3.9 mmol) in DMSO (7.5 mL) and THF (1.9 mL) sodium hydride (60 % in oil, 3.9 mmol, 156 mg) was added, and the reaction mixture was stirred at 20 °C for 15 hours. The reaction mixture was poured into 50 mL of an ice water and 1 M phosphate buffer, p*H* 7 (10 mL), stirred for one hour, the resulting precipitate was filtered off, washed with water (2 ×10 mL), methanol (10 mL), hexane (10 mL), and dried on the air to obtain a crude intermediate 1-(2-bromopyridin-4-yl)-3-[4-(dimethylamino)phenyl]propane-1,3-dione (964 mg) as an orange solid. To a suspension of this crude intermediate in THF (20 mL) hydrazine hydrate (292 µL, 300 mg, 6 mmol) was added. The reaction mixture was stirred at 70 °C for five hours, cooled and concentrated *in vacuo*. The residue was suspended in methanol (10 mL), filtered off, washed with cold methanol (2×5 mL), recrystallized from *n*-butanol (10 mL) and *N*,*N*-dimethylformamide (0.2 mL), and dried in high vacuo at 20 °C for 15 hours afforded the product 4-[3-(4-dimethylaminophenyl)-1*H*-pyrazol-5-yl]-2-bromopyridine (715 mg, 2.08 mmol, 69 % over two steps) as a light pink solid. ^1^H-NMR (400 MHz, DMSO-d_6_ + 1 % DCl) *δ* = 8.42 (d, *J*= 5.2 Hz, 1H), 8.05 (d, *J*= 1.4 Hz, 1H), 7.93 (d, *J*= 8.7 Hz, 2H), 7.87 (dd, *J*= 5.2, 1.4 Hz, 1H), 7.66 (bd, *J*= 7.8 Hz, 2H), 7.51 (s, 1H), 3.11 (s, 6H). ^13^C-NMR (100.6 MHz, DMSO + 1 % DCl) *δ* = 151.0, 145.4, 144.6 (2C), 142.5, 142.3, 127.9, 126.5 (2C), 123.2, 119.6 (2C), 119.1, 101.8, 44.2 (2C). LC MS (RP18-100Å, gradient 50 % CH_3_CN /50 % H_2_O →100 % CH_3_CN in 30 minutes), RT 4.8 minutes and mass 343.1 (100 %), 345.2 (97 %), [M+H]^+^. TLC (SiO_2_, *n*-hexane/EtOAc = 2/1) R_f_ 0.36, m.p.: 224-226 °C.

*tert*-Butyl *N*-(4-acetylphenyl)-*N*-(^2^H_3_)methylcarbamate **4b**

A mixture of *tert*-butyl *N*-(4-acetylphenyl)carbamate **3** (FW 235.28, 2.35 g, 10 mmol) and Cs_2_CO_3_ (FW 325.82, 6.52 g, 20 mmol) in diglyme (20 mL) was stirred at room temperature for ten minutes. CD_3_I (FW 144.96, d 2.33, b.p. 42 °C; 933 µL, 2.17 g, 15 mmol) was added and stirring was continued for 20 hours. The reaction mixture was quenched with 1 M phosphate buffer, p*H* 7 (20 mL), ethyl acetate (30 mL) was added, the organic phase was separated, washed with water (3 × 20 mL), dried over Na_2_SO_4_ and evaporated. The oily residue (2.67 g) was purified on silica gel (*n*-hexane/EtOAc = 3/1 R_f_ 0.38) to give 2.1 g (8.3 mmol, 83 %) of pure product as a slightly yellow oil. ^1^H NMR (400 MHz, CDCl_3_, 298K) *δ* = 7.91 (d, *J* = 8.6 Hz, 2H), 7.35 (d, *J* = 8.6 Hz, 2H), 2.57 (s, 3H), 1.47 (s, 9H). ^13^C NMR (100.6 MHz, CDCl_3_, 298K) *δ* = 197.1, 154.1, 148.0, 133.4, 128.8 (2C), 124.2 (2C), 81.1, 40.0 (sept, J_D-C_ = 21.3 Hz), 28.2 (3C), 26.5. LC MS (RP18-100 Å, gradient 50 % CH_3_CN /50 % H_2_O → 100 % CH_3_CN in 30 minutes), RT 10.0 minutes and mass 253.2 (5 %), [M+H]^+^, 238.1 (14 %), [M+H-CH_3_]^+^. 196.8 (100 %), [M+H-C_4_H_9_]^+^.

*tert*-Butyl *N*-(4-acetylphenyl)-*N*-methylcarbamate **4a**

The title compound was prepared analogously to **4b** using CH_3_I as an educt to give the compound **4a** in 94 % yield. This compound has been reported previously [[1](#_ENREF_1)], CAS [907209-80-7]. ^1^H NMR data match those reported in the literature [[1](#_ENREF_1)].

*tert*-Butyl {4-[3-(2-bromopyridin-4-yl)-3-oxopropanoyl]phenyl}methylcarbamate **5a**

To a solution of *tert*-butyl *N*-(4-acetylphenyl)-*N*-methylcarbamate **4a** (498 mg, 2.00 mmol) and methyl 2-bromopyridine-4-carboxylate **2** (518 mg, 2.4 mmol) in DMSO (5 mL) and THF (1.3 mL) sodium hydride (60 % in oil, 2.4 mmol, 96 mg) was added, and the mixture was stirred at 20 °C for 15 hours. TLC analysis of sampled 20 µL aliquot showed incompleteness of reaction (*n*-hexane/EtOAc = 3:1, carbamate R_f_ 0.5; product R_f_ 0.35). Methyl 2-bromopyridine-4-carboxylate (43 mg, 0.2 mmol) and sodium hydride (60 % in oil, 0.4 mmol, 16 mg) were added additionally, and the stirring was continued for 15 hours. The reaction mixture was poured into ice water (20 mL) and 1 M phosphate buffer p*H* 7 (10 mL), stirred for 30 minutes, and extracted with chloroform (3 × 10 mL). The solution was washed with brine, dried over Na_2_SO_4_ and concentrated *in vacuo*. The resulting oil was purified on 90 g silica gel (*n*-hexane/EtOAc = 3:1, R_f_ 0.39) to afford the *tert*-butyl {4-[3-(2-bromopyridin-4-yl)-3-oxopropanoyl]phenyl}methylcarbamate (580 mg, 1.34 mmol, 67 %) as a yellow glass. ^1^H NMR (400 MHz, CDCl_3_, 298K) *δ* = 8.53 (d, *J* = 5.1 Hz, 1H), 7.97 (m, 3H), 7.73 (dd, *J* = 5.1, 1.4 Hz, 1H), 7.44 (m, 2H), 6.80 (s, 1H), 3.34 (s, 3H), 1.50 (s, 9H). ^13^C NMR (100.6 MHz, CDCl_3_, 298K) *δ* = 187.6, 178.7, 153.8, 150.7, 148.3, 144.8, 142.9, 130.5, 127.9 (2C), 124.9, 124.2 (2C), 119.3, 93.9, 81.1, 36.6, 28.1 (3C). LC-MS (RP18-100 Å, gradient 50 % CH_3_CN /50 % H_2_O → 100 % CH_3_CN in 30 minutes), RT 24.1 min and mass 433.1 (100%), 435.3 (98%) [M+H]^+^.

*tert*-Butyl {4-[3-(2-bromopyridin-4-yl)-3-oxopropanoyl]phenyl}(^2^H_3_)methylcarbamate **5b**

The title compound was prepared analogously to **5a** using **4b** as an educt to give the compound **5b** in 65 % yield. ^1^H NMR (400 MHz, CDCl_3_, 298K) *δ* = 8.52 (d, *J* = 5.1 Hz, 1H), 7.96 (m, 3H), 7.72 (dd, *J* = 5.1, 1.4 Hz, 1H), 7.43 (m, 2H), 6.79 (s, 1H), 1.50 (s, 9H). ^13^C NMR (100.6 MHz, CDCl_3_, 298K) *δ* = 187.6, 178.7, 153.8, 150.7, 148.2, 144.9, 142.9, 130.5, 127.9 (2C), 124.9, 124.1 (2C), 119.3, 93.9, 81.2, 35.9 (sept, J_D-C_ = 21.2 Hz), 28.1 (3C). LC-MS (RP18-100 Å, gradient 50 % CH_3_CN /50 % H_2_O → 100 % CH_3_CN in 30 minutes), RT 24.2 min and mass 436.3 (100%), 438.3 (98%) [M+H]^+^. TLC (SiO_2_, *n*-hexane/EtOAc = 3:1) R_f_ = 0.39.

*tert*-Butyl { 4-[5-(2-bromopyridin-4-yl)-1*H*-pyrazol-3-yl]phenyl }(^2^H_3_)methylcarbamate **6b**

To a solution of the *tert*-butyl {4-[3-(2-bromopyridin-4-yl)-3-oxopropanoyl]phenyl}(^2^H_3_)methylcarbamate **5b** (873 mg, 2 mmol) in THF (10 mL) was added hydrazine monohydrate (194 µL, 200 mg, 4 mmol). After being stirred at 60 °C for 15 hours the reaction mixture was cooled to room temperature, concentrated *in vacuo* and evaporated with methanol (10 mL). The residue (900 mg) was purified on silica gel (CHCl_3_/MeOH = 95:5, R_f_ 0.31) to give 830 mg (1.9 mmol, 96 %) of the product as a beige foam. ^1^H NMR (400 MHz, CDCl_3_, 298K) *δ* = 11.68 (bs, 1H), 8.36 (d, *J* = 5.2 Hz, 1H), 7.88 (s, 1H), 7.63 (d, *J* = 5.2 Hz, 1H), 7.54 (d, *J* = 8.5  Hz, 2H), 7.31 (d, *J* = 8.5  Hz, 2H), 6.84 (s, 1H), 1.50 (s, 9H). ^13^C NMR (100.6 MHz, CDCl_3_, 298K) *δ* = 154.7, 150.2, 147.0, 145.9, 144.0, 142.5, 142.4, 125.7 (3C), 125.5 (2C), 124.0, 119.0, 100.9, 80.9, 36.4 (sept, J_D-C_ = 21.2 Hz), 28.3 (3C). LC-MS (RP18-100 Å, gradient 50 % CH_3_CN /50 % H_2_O → 100 % CH_3_CN in 30 minutes), RT 15.3 min and mass 432.3 (100%), 434.3 (98%) [M+H]^+^.

*tert*-Butyl { 4-[5-(2-bromopyridin-4-yl)-1*H*-pyrazol-3-yl]phenyl }methylcarbamate **6a**

The title compound was prepared analogously to **6b** using **5a** as an educt to give the compound **6a** in 96 % yield. ^1^H NMR (400 MHz, CDCl_3_, 298K) *δ* = 8.36 (d, *J* = 5.2 Hz, 1H), 7.88 (m, 1H), 7.63 (dd, *J* = 5.2, 1.4 Hz, 1H), 7.54 (d, *J* = 8.5  Hz, 2H), 7.32 (d, *J* = 8.5  Hz, 2H), 6.84 (s, 1H), 3.29 (s, 3H), 1.50 (s, 9H). ^13^C NMR (100.6 MHz, CDCl_3_, 298K) *δ* = 154.7, 150.3, 147.3, 145.9, 143.9, 142.6 (2C), 126.0, 125.6 (4C), 124.0, 119.0, 100.8, 80.9, 37.1, 28.3 (3C). LC-MS (RP18-100 Å, gradient 50 % CH_3_CN /50 % H_2_O → 100 % CH_3_CN in 30 minutes), RT 15.5 min and mass 429.3 (100%), 431.3 (98%) [M+H]^+^. TLC (SiO_2_, CHCl_3_/MeOH = 95:5) R_f_ = 0.31.

4-[5-(2-Bromopyridin-4-yl)-1*H*-pyrazol-3-yl]-*N*-(^2^H_3_)methylaniline **7b**

To a solution of *tert*-butyl {4-[5-(2-bromopyridin-4-yl)-1*H*-pyrazol-3-yl]phenyl}(^2^H_3_)methylcarbamate **6b** (810 mg, 1.87 mmol) in CH_2_Cl_2_ (15 mL) trifluoroacetic acid (2 mL, 2.96 g, 26 mmol) was added. The mixture was stirred at room temperature for 15 hours and concentrated *in vacuo*. 1 M phosphate buffer, p*H* 7 (20 mL) was added, the resulting precipitate was filtered off, washed with water (2 × 10 mL) and air dried for 15 hours to give 559 mg (1.68 mmol, 90 %) of product as a beige-colored solid with HPLC purity 99 %+ (sample 0.5 mg/mL in CH_3_CN; 5 µL injection volume; column: Eurospher RP18 100Å, 5 µm, 250×4.6 mm, solvents: water (+0.1 % TFA, A) and CH_3_CN (+0.1 % TFA, B), gradient: B 0 % ®100 % in 30 minutes, detector UV 260 nm, peak RT 17.7 minutes). ^1^H NMR (400 MHz, TFA-d_1_, 298K) *δ* = 8.46 (d, *J* = 6.4 Hz, 1H), 8.31 (s, 1H), 8.12 (d, *J* = 6.4 Hz, 1H), 7.70 (d, *J* = 8.6 Hz, 2H), 7.43 (d, *J* = 8.6 Hz, 2H), 7.22 (s, 1H). ^13^C NMR (100.6 MHz, TFA-d_1_, 298K) *δ* = 151.0, 148.9, 146.4, 146.2, 139.3, 137.2, 131.5, 131.0 (2C), 130.5, 125.1 (2C), 124.1, 108.1, 40.0 (m, J_D-C_ = 21.1 Hz). LC MS (RP18-100 Å, gradient 0 % CH_3_CN /100 % H_2_O → 100 % CH_3_CN in 30 minutes), RT 17.7 minutes and mass 329.1 (98 %), 331.1 (100 %), [M+H]^+^. TLC (SiO_2_, *n*-hexane/EtOAc = 1/1) R_f_ 0.40, m.p.: 224-225 °C.

4-[5-(2-Bromopyridin-4-yl)-1*H*-pyrazol-3-yl]-*N*-methylaniline **7a**

The title compound was prepared analogously to **7b** using **6a** as an educt to give the compound **6a** in 74 % yield as a beige‑colored solid with HPLC purity 98.3 % (sample 0.1 mg/mL in CH_3_CN; 5 µL injection volume; column: Eurospher RP18 100 Å, 5 µm, 250 × 4.6 mm, solvents: water (+0.1 % TFA, A) and CH_3_CN (+0.1 % TFA, B), gradient: B 0 % → 100 % in 30 minutes, detector UV 254 nm, peak RT 17.7 min). ^1^H NMR (400 MHz, DMSO-d_6_ + 1 % DCl, 313K) *δ* = 8.41 (d, *J* = 5.2 Hz, 1H), 8.04 (d, *J* = 1.1 Hz, 1H), 7.93 (d, *J* = 8.6 Hz, 2H), 7.86 (dd, *J* = 5.2, 1.4 Hz, 1H), 7.57 (d, *J* = 8.6 Hz, 2H), 7.48 (s, 1H), 2.92 (s, 3H). ^13^C NMR (400 MHz, DMSO-d_6_ + 1 % DCl, 313K) *δ* = 150.8, 145.5, 145.0, 142.2, 142.1, 138.7, 129.0, 126.5 (2C), 123.1, 121.6 (2C), 119.0, 101.9, 35.0. LC MS (RP18-100 Å, gradient 0 % CH_3_CN /100 % H_2_O → 100 % CH_3_CN in 30 minutes), RT 17.7 minutes and mass 329.1 (98 %), 331.1 (100 %), [M+H]^+^. TLC (SiO_2_, *n*-hexane/EtOAc = 1/1) R_f_ 0.40, m.p.: 225-226 °C.

**Preparation and characterization of recombinant αSYN fibrils and hTau46 fibrils**

Expression and purification of recombinant wild‑type αSYN was performed as previously described [[2](#_ENREF_2)]. Briefly, pET‑5a/α‑synuclein (136TAT) plasmid (wt plasmid provided by Philipp Kahle, Hertie Institute for Clinical Brain Research, Tübingen; 136-TAC/TAT‑Mutation provided by Matthias Habeck, LMU Munich) was used to transform *E. coli* BL21(DE3)pLys cells (Novagen, Madison, WI, USA), and expression was induced with isopropyl‑β‑D‑thiogalactopyranose (IPTG, Peqlab, Erlangen, Germany). Cells were lysed by boiling after heat inactivation of proteases. After centrifugation, the supernatant was filtered through a Filtropur S 0.2 μm filter (Sarstedt, Nümbrecht, Germany), loaded on a HiTrap Q HP anion exchange column (5 mL, GE Healthcare, Munich, Germany) and eluted with a linear gradient of 25 mM to 500 mM NaCl. Fractions containing αSYN were pooled and further purified by gel filtration via a Superdex 75 prep‑grade column (25 mL, GE Healthcare). The protein concentration was adjusted to 1 mg/mL in 50 mM Tris‑HCl, p*H* 7.0. After freezing in liquid nitrogen, the protein was stored at -80 °C. Fibrilization was induced by constant agitation at high protein concentrations as described previously [[3](#_ENREF_3), [2](#_ENREF_2)]. Briefly, 50 µM αSYN in 50 mM Tris‑HCl containing 100 mM NaCl and 0.02 % NaN_3_, p*H* 7.0, was incubated for 96 hours at 37 °C and 1400 rpm using an Eppendorf Thermomixer Comfort (Eppendorf, Hamburg, Germany). To purify the fibrils from nonaggregated monomeric αSYN, the fibril preparations were ultracentrifuged at 135,000 × g at 4 °C for 30 minutes in a Beckman Optima Max-XP centrifuge (Beckman Coulter, Krefeld, Germany). The pellet was resuspended in aggregation buffer, and the concentration was determined using a BCA assay. After freezing in liquid nitrogen, fibrils were stored at ‑80 °C. Recombinant hTau46 was purchased lyophilized from Bio-Techne GmbH (Wiesbaden, Germany) and was solved in 50 mM Tris‑base, p*H* 7.0. Fibrillization was induced by heparin under constant agitation, as described previously [[4](#_ENREF_4)]. Briefly, 10 µM hTau46 was incubated in presence of 0.02 % NaN_3_ and 0.03 mg/mL heparin (sodium salt, Sigma Aldrich, Taufkirchen, Germany) for 72 h at 37 °C and 1,000 rpm. Fibril formation was verified using Thioflavin (Th)T‑fluorescence and sucrose density centrifugation. ThT‑fluorescence was measured using a LS55 Luminescence Spectrometer (Perkin Elmer, Hamburg, Germany) with 0.5 μM protein and 20 μM ThT (Sigma Aldrich, Taufkirchen, Germany) in 50 mM Tris‑HCl, p*H* 7.5. Spectra were recorded at wavelengths ranging from 460 nm to 560 nm. A continuous sucrose gradient assay was performed as described previously [[3](#_ENREF_3), [2](#_ENREF_2)]. Briefly, solutions containing 50 mM Tris‑Base, p*H* 7.5, 0.1 % NP-40 (Roche, Mannheim, Germany) and sucrose (10 %, 20 %, 30 %, 40 %, 50 % and 60 %, respectively) were filled into a 4 mL 11 × 60 mm polyallomer tube (Beckman Coulter), with 200 μL of the 60 % sucrose solution first loaded into the bottom, followed by 400 μL of the 50 % solution to the 10 % sucrose solution. Finally, 200 μL of 5 μM protein in 1x TBS (p*H* 7.5) containing 0.1 % NP-40 was loaded on the top of the gradient. Ultracentrifugation at 40,000 x g and 4 °C for one hour was performed in a Sorvall WX Ultra 90 centrifuge using a Sw60Ti rotor (Beckman Coulter). The resulting continuous gradients were fractionated in volumes of 200 μL. Twenty microliters per fraction were analyzed by denaturing SDS‑PAGE and Western blot analysis using a monoclonal antibody against human αSYN (4B12, Biolegend, San Diego, CA, USA), and human Tau46, respectively (Cell Signaling Technology, Leiden, Netherlands).

## Preparation and characterization of Aβ_1‑42_ fibrils

Aβ_1‑42_ fibril production was adapted from Bagchi *et al.* [[5](#_ENREF_5)]. One milligram of synthetic lyophilized human Aβ_1‑42_ peptide with >90 % purity (EMC Microcollections, Tuebingen, Germany) was dissolved in 44.3 µL of DMSO. Deionized water (820 µL) and 1 M Tris‑HCl (22.2 µL, p*H* 7.6) were added to reach a final monomer concentration of 250 µM (1.13 mg/mL). Aggregation was induced by shaking at 800 rpm in an Eppendorf Thermomixer for 65 hours at 37 °C. Fibrils were sonicated in a water bath (Elmasonic S 60 H, Elma Schmidbauer GmbH, Singen, Germany) at 37 °C for three minutes. The presence of β‑helical secondary structure was confirmed via an increase in ThT (AB 137040, abcr GmbH, Karlsruhe, Germany) fluorescence determined by obtaining an emission spectrum. To this end, Aβ_1‑42_ was diluted in 50 mM glycine/NaOH, p*H* 8.5, with 10 µM ThT to a final concentration of 2.5 µM as described previously [[6](#_ENREF_6), [7](#_ENREF_7)].

## Negative staining transmission electron microscopy

Different fibril suspensions were placed directly onto a glow‑discharged electron microscopy (EM) grid. After adsorption, the grid was washed in double distilled water and negatively stained with 1 % uranyl acetate. The grids were examined using a Zeiss LIBRA 120 transmission electron microscope (Carl Zeiss, Oberkochen, Germany) operating at 120 kV.

## Fibril concentration determination

To avoid radioligand depletion in binding experiments, the radioligand needs to be added in access. The concentration of human recombinant αSYN, hTau46 and Aβ_1-42_ fibrils at which ≤ 10 % of the added ligand binds to the target molecule was determined for saturation and competition experiments, according to [[8](#_ENREF_8)]. Briefly, decreasing fibril concentrations were incubated with 1 nM [^3^H]MODAG-001 for two hours at 37 °C. Washing and harvesting of fibrils was performed as described in the next paragraph. Radioactivity converted into % total bound/added radioligand was plotted against increasing fibril concentrations.

## Binding assays – filtration and read-out

Plates covered by removable sealing tape (PerkinElmer, Waltham, MA, USA) were incubated on a shaker (MaxQ™ 6000, orbit diameter 1.9 cm, Thermo Fisher Scientific Inc., Marietta, OH, USA) at 45 rpm for two hours at 37 °C. After incubation, bound and free radioligands were separated by vacuum filtration through glassfiber filtermat B (PerkinElmer) using a filtermat harvester (PerkinElmer). To harvest plates containing αSYN and Aβ_1-42_ fibrils, the filtermat was additionally incubated with 5 mg/mL polyethylenimine for 30 minutes at 4 °C prior to harvesting. The filter was washed three times with 100 mL (approximately 1 mL/well) of ice cold incubation buffer and subsequently dried in a microwave for 2.5 minutes at medium power. Melt on scintillator sheets (MeltiLex™ B/HS, PerkinElmer) were melted into the filter using a heating plate set to 120 °C. After hardening at room temperature, the filter was sealed in a plastic sample bag (PerkinElmer). Accumulation of tritium was immediately counted in a Wallac MicroBeta® TriLux liquid scintillation counter (PerkinElmer). Radioactivity was plotted against ^3^H-labeled compound concentration. Data points were fitted using nonlinear regression analysis in GraphPad Prism (GraphPad Software, Inc., version 7.03, La Jolla (CA), USA). Saturation binding experiments were performed as three independent experiments using three technical replicates for TB and NSB in each experiment.

## Radiosynthesis of [^11^C]MODAG‑001 and (d_3_)‑[^11^C]MODAG‑001

[^11^C]CO_2_ was produced on a PETtrace 860 cyclotron (GE Healthcare, Uppsala, Sweden) and reacted to [^11^C]MeI using a Tracerlab FX MeI module (GE Healthcare) according to the manufacturer’s recommendations. A Tracerlab FX M module (GE Healthcare) was used for radiomethylation, HPLC purification and formulation. Depending on the required tracer amount and A_m_, either direct methylation (high A_m_) or reductive methylation (high yield) was applied. For direct methylation, the alkylating reagent was bubbled through a solution of 1 mg of precursor **7a** (nondeuterated or deuterated) in 500 µL of DMSO at 18 °C. The methylation reaction was performed for five minutes at 110 °C. For reductive methylation, the alkylating reagent was bubbled through a solution of 5 mg of Me_3_NO and 1 mg of precursor in 350 µL of diethyl formamide at ‑25 °C. The mixture was heated to 60 °C for three minutes and subsequently cooled to 40 °C. NaBH_3_CN (7.6 mg) in a mixture of 60 µL of diethyl formamide and 1.2 mL of 100 mM citrate‑phosphate buffer (p*H* 5) was added and heated to 100 °C for five minutes. After labeling, the reaction was subjected to semipreparative HPLC on a Synergi Max‑RP column (4 µm, 80 Å, 250 mm × 10 mm, Phenomenex, Aschaffenburg, Germany) with 55 % MeCN in water at a flow rate of 6 mL/min. The HPLC fraction containing the product was diluted with 30 mL of water and loaded onto a Sep‑Pak C8 Plus Light cartridge (Waters, Milford, MA, USA). The product was eluted with 0.3 mL of ethanol and diluted with 3 mL of PBS.

Quality control and measurement of A_m_ were performed on a 1260 Infinity HPLC system (Agilent Technologies, Waldbronn, Germany). An analytical Luna Phenyl‑Hexyl column (5 µm, 100 Å, 300 mm × 4.6 mm, Phenomenex) was used for separation, with 30 % MeCN in 0.1 % aqueous trifluoroacetic acid at an isocratic flow rate of 1.5 mL/min. The carrier content was calculated from linear regression of a calibration curve of the nonradioactive standard molecule.

**Fibril inoculation into the rat brain**

Rats were allowed to adapt for two weeks in the animal facility before fibril injection. Three adult female rats were anaesthetized with an intraperitoneal (i.p.) injection of a mixture of fentanyl (0.005 mg/kg), midazolam (2 mg/kg), and medetomidine (0.15 mg/kg) at 1 mL/kg. The head of each rat was shaved, and the animal was placed into a stereotaxic frame. A central incision was made to expose the bregma and lambda. A 5 µL syringe needle (Hamilton Company, Reno (NV), USA) was enclosed with a glass capillary (inner diameter, 50 µm, Hilgenberg GmbH, Malsfeld, Germany). αSYN fibrils (4 µL, 30 µM) were injected using a stereotaxic injector (Stoelting, Wood Lane, IL, USA) through a drill hole into the right striatum (ML= -3.2 mm, AP = 0.0 mm, DV = -4.8 mm) according to the stereotaxic atlas of Paxinos and Watson [[9](#_ENREF_9)][. Based on the volume of the right striatum (0.04 cm^3^), the concentration of inoculated fibrils was calculated as 2.8 µM. As a negative control, 4 µL of vehicle (50 mM Tris base, 100 mM sodium chloride, 0.02 % sodium azide, p*H* 7.0) were injected into the contralateral striatum. A total fibril volume of 4 µL was obtained by the injection of 0.4 µL every 60 seconds. To allow for diffusion of the fibrils into the tissue, the needle was left in this position for five minutes. Before slowly retracting the needle from the brain (0.5 mm/10 s), it was withdrawn 0.2 mm (DV) and maintained for another minute. The incision was closed, and a subcutaneous antidote injection of atipamezole (0.75 mg/kg) and flumazenil (0.2 mg/kg) was administered.

**PET data acquisition and analysis**

Dynamic PET data were acquired for 60 minutes and divided into 39 time frames (12 × 5 s, 6 × 10 s, 6 × 30 s, 5 × 60 s and 10 × 300 s). A transmission measurement using a cobalt-57 point source was performed for 13 minutes after PET data acquisition for attenuation correction. The data were reconstructed into a dynamic PET image using the reconstruction algorithm OSEM3D.

Whole body mouse PET scans were coregistered to the whole body MR scan, which was sequentially acquired after the PET scan, and the volumes of interest (VOIs) of the lung, liver, heart, brain, and kidneys were drawn by hand based on the MR anatomical information using PMOD software (version 3.2; PMOD Technologies, Zürich, Switzerland). VOIs of different brain regions were extracted using the mouse or rat brain atlas provided by PMOD [[10-12](#_ENREF_10)], and tissue time activity curves (TACs) were calculated and are expressed as standardized uptake values (SUVs), which were calculated as follows: SUV(t) = radioactivity concentration (kBq/mL of the organ)/(injected dose [kBq]/body weight [g]). For inoculated rats, an additional VOI was placed over the right striatum using the isocontour tool adjusted to 70 % of the peak counts of hot spots, which was copied to the left control striatum with the mirroring tool. Distribution volume ratio 1 (DVR-1) values were calculated from individual TACs from 40 to 60 minutes, using the vehicle-injected striatum as a reference region, with the following formula: activity in fibril inoculated right striatum/activity in vehicle injected left striatum) ‑ 1. The mean SUV from 40 to 60 minutes between the right, fibril inoculated striatum and contralateral, vehicle injected striatum was tested for significance using a two sided t test. All fibril-inoculated rats were sacrificed under CO_2_ after PET acquisition and subsequently perfused with 100 mL of cold PBS. Brains were dissected and immediately frozen in 2-methybutane on dry ice.

## Thioflavin S staining

Coronal cryosections (30 µm) of fibril‑inoculated rats were air‑dried at room temperature and stored at -20 °C until further use. For thioflavin S staining, sections were allowed to thaw for 30 minutes at room temperature, fixed for 15 minutes in 4.5 % formaldehyde, washed three times for five minutes each in PBS and rinsed for one minute in 60 % and then 80 % ethanol. Sections were subsequently incubated for 15 minutes in 0.1 % thioflavin S (Sigma‑Aldrich Chemie GmbH) dissolved in 80 % ethanol. After one‑minute rinses in 80 % ethanol and 60 % ethanol and two washes in PBS for five minutes each, the slides were mounted with Eukitt quick‑hardening mounting medium (Sigma-Aldrich Chemie GmbH) and allowed to dry overnight. Whole‑brain images of the stained sections were captured using the FITC filter settings (excitation, 460 nm‑500 nm; emission, 512 nm‑542 nm) of a Leica DMi8 microscope interfaced with Leica LAS X software (Leica Microsystems CMS GmbH, Wetzlar, Germany).

## Metabolite analysis

A 400 µL blood sample was collected by heart puncture five or 15 minutes after tracer injection. The blood was centrifuged at 17,000 x g and 4 °C for two minutes, and the plasma was transferred to a 1.5 mL reaction tube on ice for further analysis. After blood collection, mice were transcardially perfused (10 mL/min) with 20 mL of ice‑cold PBS, and the brain of each mouse was removed from the skull. The brain was transferred to a glass tube (2 mL Dounce tissue grinder set, Sigma Aldrich Chemie GmbH) containing 0.5 mL of ice‑cold PBS, sequentially homogenized using large and small clearance pestles and transferred to a 1.5 mL reaction tube on ice for further analysis. Plasma or brain homogenate was mixed 1:1 with acetonitrile and briefly centrifuged. After two minutes of incubation on ice, each mixture was centrifuged at room temperature for 1.5 minutes at 12,000 x g (MiniSpin®, Eppendorf AG) to remove precipitated proteins. The supernatant was subjected to reversed‑phase HPLC on a Luna Phenyl Hexyl column (5 µm, 100 Å, 300 mm x 4.6 mm, Phenomenex) equipped with a radioactivity detector with 0.1 % trifluoroacetic acid in water/acetonitrile (70:30) at an isocratic flow rate of 1.5 mL/min.

For data analysis, the median of ten consecutive data points was calculated to remove noise; data were corrected for radioactive decay to time zero, defined as start of the HPLC of the five minute brain sample. This time zero radioactivity was plotted against time using Origin software (OriginLab Corporation, version 9.1G, Northampton, MA, USA). The area under the curve was calculated in % using the peak analyzer function and scaled up that the peaks add up to 100 %. The area under the curve was transformed into radioactivity values using a standard of [^11^C]MODAG‑001 with known radioactivity also corrected for decay and expressed as % injected radioactivity dose (%ID) per gram of brain tissue or per plasma in one mL of blood.

## MRI

Whole‑body anatomical MR images of the mice were acquired on a 7T small animal MRI scanner (Bruker BioSpin GmbH, Ettlingen, Germany) following PET data acquisition. Anesthetization of the animals was maintained with 1.5 % isoflurane evaporated in 100 % oxygen at a flow rate of 0.8 L/min. A rat whole‑body coil (Bruker BioSpin GmbH) was used for MR measurements in mice. After the mouse was positioned in the center of the FOV, a Turbo RARE T2 (TR/TE 800 ms/35.1 ms; FoV, 37.4 mm × 85.8 mm × 22.8 mm; matrix, 144 × 256 × 92) sequence was used to acquire the anatomical image of the body.

***In vitro* autoradiography**

The defrosted human sections were preincubated in incubation buffer (50 mM Tris‑base, 10 % ethanol, 0.05 % Tween80, p*H* 7.4) for 25 minutes at room temperature. Subsequently, the sections were incubated in 3 nM [^3^H]MODAG‑001 for total binding (TB). Nonspecific binding (NSB) of the radiotracer was determined by coincubation of consecutive slices with 50 μM nonradioactive MODAG‑001 dissolved in DMSO (final concentration 0.5 %). After incubation for 60 minutes at room temperature, the sections were washed three times for ten minutes in ice‑cold incubation buffer and dipped for three seconds in deionized water. The sections were dried overnight in a desiccator filled with powdered paraformaldehyde at room temperature. Slides were then exposed to phosphor imaging plates (BAS-IP TR2025, Fuji Imaging Plate, VWR, Denmark) with a tritium standard (American Radiolabeled Chemicals, St. Louis, MO, USA) for seven days and scanned in a phosphor imager (BAS‑5000, FUJIFILM Life Science, Stamford, CT, USA), and the images were displayed with Image Gauge version 4.0 (Science Lab 2001, FUJIFILM Life Science, Stamford, CT, USA). For data quantification, rectangular regions of interest (ROIs) were placed over the tritium standards to obtain a standard curve for the quantification of radioactivity concentrations. Next, four rectangular ROIs per slice were placed into the cortex of each case to calculate TB and NSB. Specific binding (SB) values were calculated by subtracting the calculated NSB values from the TB values interpolated from the standard curve and converted to pmol/mg.

## Continuous sucrose gradient centrifugation

For continuous sucrose gradient centrifugation 10 % (w/v) brain homogenates were prepared in 50 mM Tris‑base, 175 mM NaCl, 1 mM MgCl_2_, 0.1 mM PMSF, 1 mM NEM, 0.1 % Nonidet P-40 Substitute (Sigma-Aldrich, Taufkirchen, Germany), protease inhibitor (cOmplete, Mini, EDTA-free Protease Inhibitor Cocktail, Hoffmann-La Roche, Basel, Switzerland), p*H* 7.5. Aliquots of the homogenate were frozen in liquid nitrogen and stored at -80 °C. Total protein concentration was determined by BCA assay (Sigma-Aldrich, Taufkirchen, Germany). For the sucrose gradient analysis 50 µL of the homogenate were used. To this end, the brain homogenate was thawed on ice and diluted in 50 mM Tris‑base, 175 mM NaCl, 0.1 % *N*-lauroylsarcosine sodium salt (sarcosyl), 0.1 % Nonidet P-40 Substitute (Sigma-Aldrich), p*H* 7.5. The samples were agitated at 1,200 rpm (ThermoMixer C, Eppendorf) and 4 °C for 30 minutes. Sucrose gradients were prepared in 4-mL 11 × 60 mm polyallomer tube (Beckman Coulter, Krefeld, Germany). 200 µL of 60 % sucrose solution in 50 mM Tris‑base, 0.1 % sarcosyl, p*H* 7.5 were pipetted into the tube followed by 400 µL of 50, 40, 30, 20 and 10 % sucrose solutions in 50 mM Tris‑base, 0.1 % sarcosyl, p*H* 7.5. Then, 200 µL of the homogenate samples were pipetted as uppermost layer onto the gradient. The samples were ultracentrifuged in a SW 60 Ti rotor (Beckman Coulter, Krefeld, Germany) at 40,000 rpm and 4 °C for one hour. Twelve fractions of 200 µL each were collected from the top to the bottom of each tube and subjected to trichloroacetic acid (TCA) precipitation (10 % TCA) overnight at -20 °C. After thawing, samples were centrifuged at 25,000 g and 4 °C for 15 minutes. The precipitates were washed once with acetone (-20 °C), centrifuged at 25,000 × g and 4 °C for 15 minutes, and finally resuspended in Laemmli sample buffer. Samples were boiled at 96 °C for five minutes and subjected to SDS-PAGE and Western Blotting. A primary antibody against αSYN (4B12, BioLegend, San Diego, CA, USA), a secondary HRP‑coupled antibody (Cell Signaling Technology, Inc., Danvers, MA, USA), and Clarity ECL substrate (Bio**‑**Rad Laboratories, Hercules, CA, USA) were used for protein detection. Signals were quantified with ImageJ (US National Institutes of Health, Bethesda, MD, USA).

**Supplemental Results**

**Fibril characterization**

αSYN fibrils were straight and arranged in bundles with an average length of 152.5 ± 76.6 nm (n = 135). In contrast, hTau46 fibrils showed less clustering or stacking and an average length of 453.4 ± 261.9 nm (n = 154). Aβ_1‑42_ fibrils had an average length of 139.4 ± 77.3 nm (n = 71) and were arranged mainly in clusters (Supplemental Fig. S 2).

# Supplemental figures

**
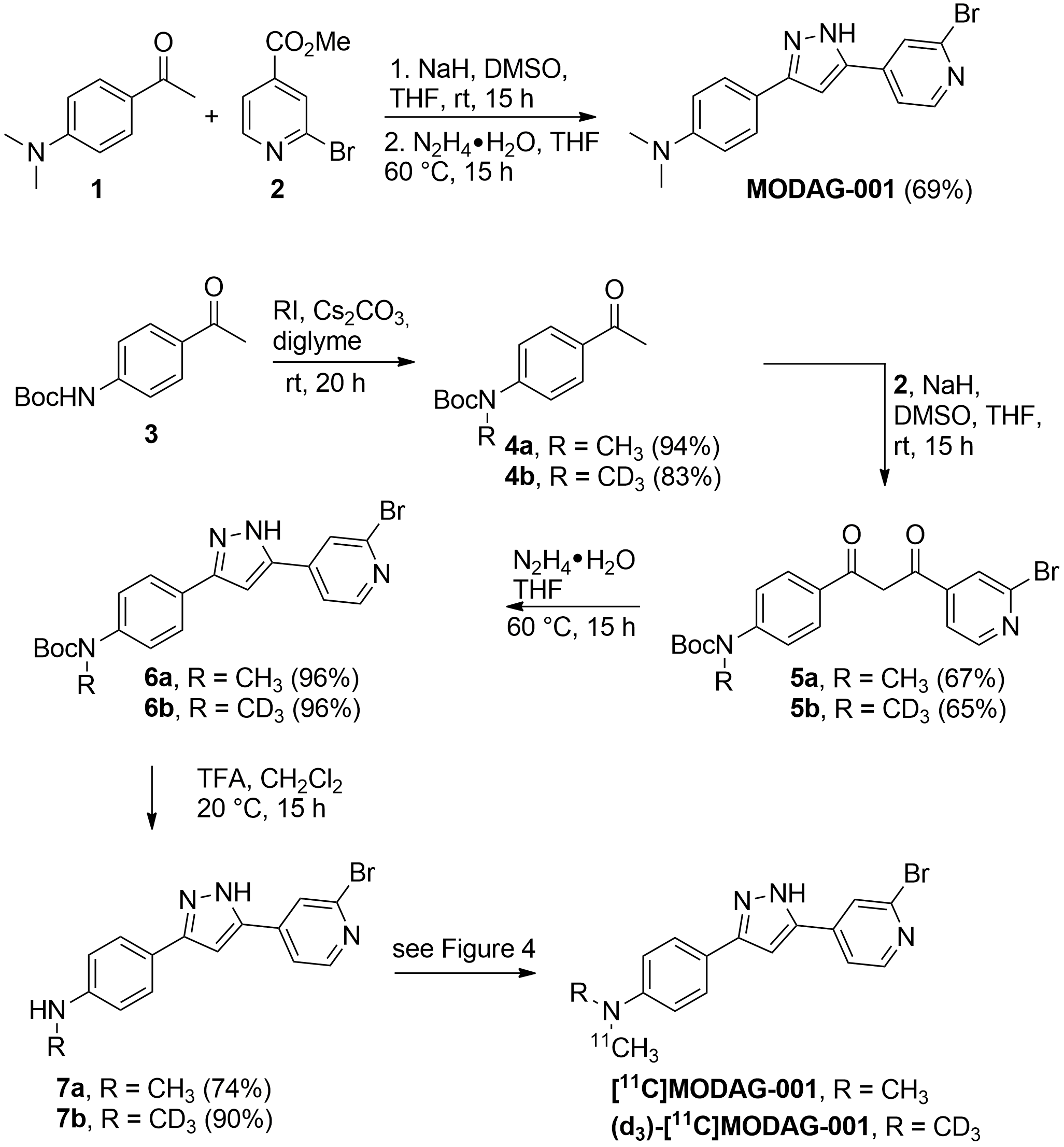
**

Supplemental Fig. S 1 Synthesis of MODAG‑001 and (d_3_)‑MODAG‑001


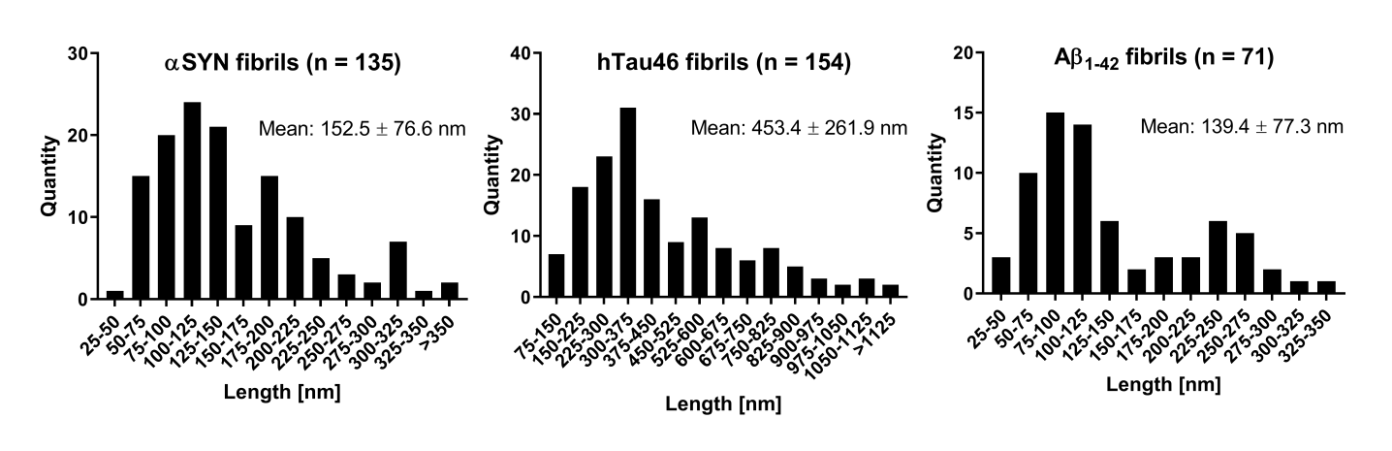


Supplemental Fig. S 2 Quantification of αSYN (a), hTau46 (b) and Aβ_1-42_ (c) fibril length using negative stain electron microscopy


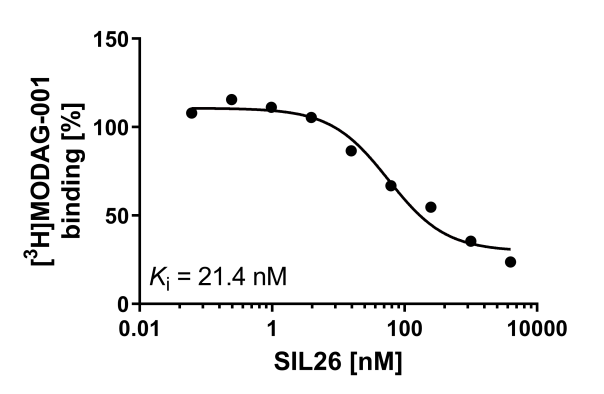


Supplemental Fig. S 3 SIL26 competition assay using 1 nM [^3^H]MODAG‑001. Non‑linear regression analysis revealed a *K*_i_ of 21.4 nM


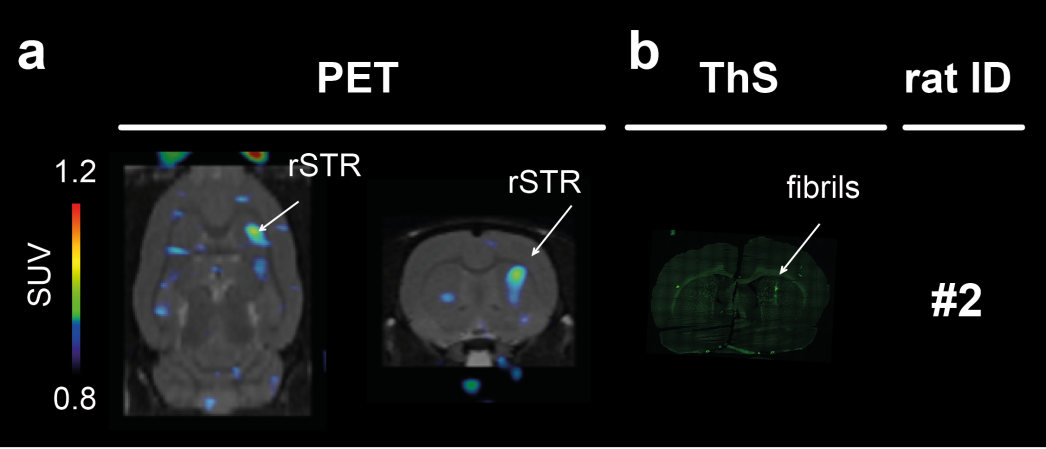


**Supplemental Fig. S 4:** *In vivo* binding of (d_3_)‑[^11^C]MODAG‑001 in the α‑synuclein‑inoculated rat #2 shown in figure 7 of the manuscript (a). Coronal and transversal PET images are shown using a SUV of 0.8 to 1.2 of the PET images to remove the unspecific signal for a better visualization of the specific binding signal. Images are summed up from 2.5 to 60 minutes. Thioflavin S staining (b) confirmed the location of αSYN fibrils (white arrow) in the right striatum of the fibril inoculated rat.


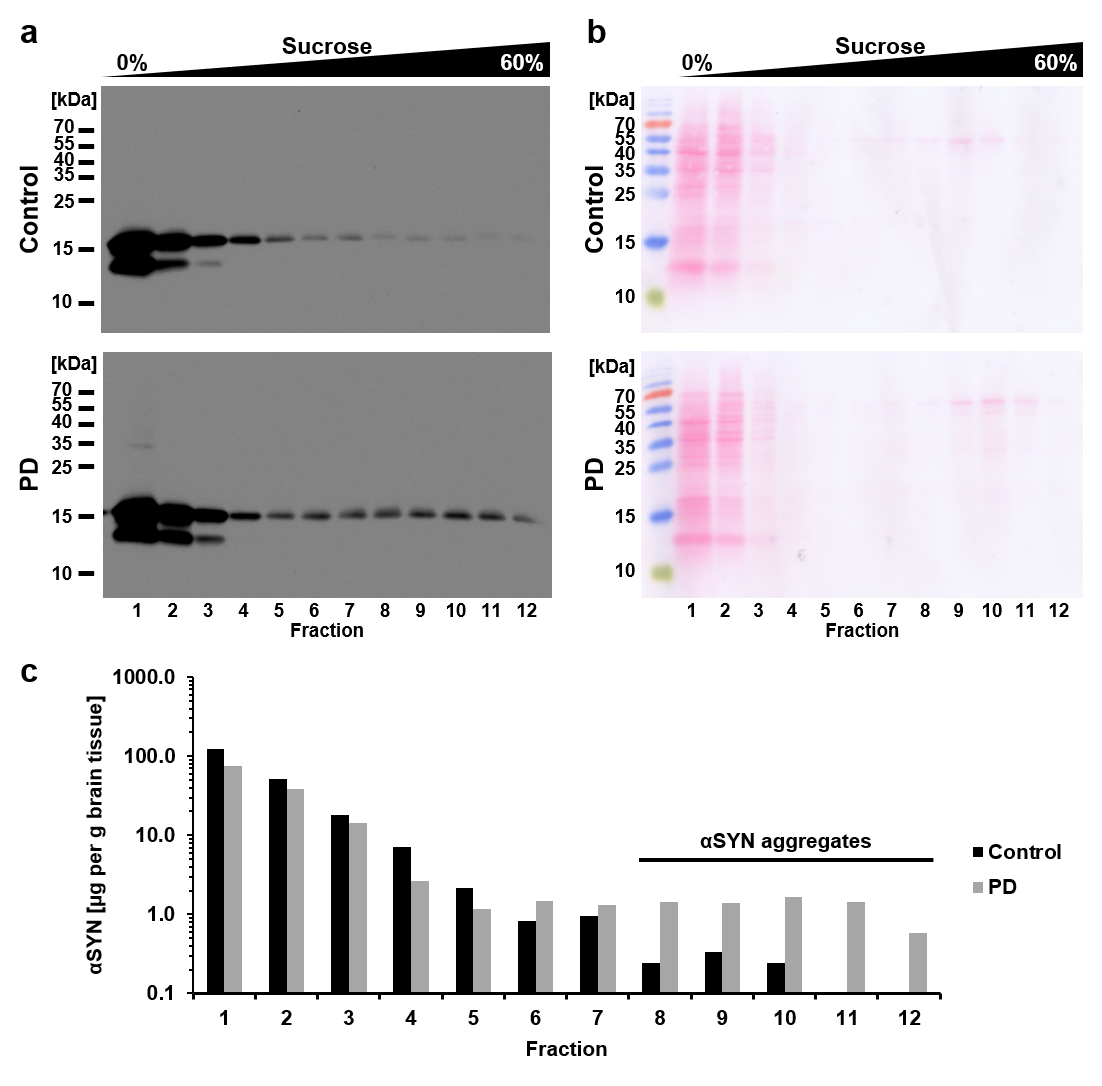


**Supplemental Fig. S 5** Quantification of the amount of aggregated αSYN in a PD brain using sucrose gradient centrifugation. αSYN was detected by Western blotting using the 4B12 antibody (a) and quantified (c). Total protein was stained with Ponceau S (Carl Roth, Karlsruhe, Germany) (b)

**References**

1. Tago T, Furumoto S, Okamura N, Harada R, Ishikawa Y, Arai H et al. Synthesis and preliminary evaluation of 2-arylhydroxyquinoline derivatives for tau imaging. J Labelled Comp Radiopharm. 2014;57(1):18-24. doi:10.1002/jlcr.3133.

2. Maurer A, Leonov A, Ryazanov S, Herfert K, Kuebler L, Buss S et al. (11) C Radiolabeling of anle253b: a Putative PET Tracer for Parkinson's Disease That Binds to alpha-Synuclein Fibrils in vitro and Crosses the Blood-Brain Barrier. ChemMedChem. 2019. doi:10.1002/cmdc.201900689.

3. Deeg AA, Reiner AM, Schmidt F, Schueder F, Ryazanov S, Ruf VC et al. Anle138b and related compounds are aggregation specific fluorescence markers and reveal high affinity binding to alpha-synuclein aggregates. Biochim Biophys Acta. 2015;1850(9):1884-90. doi:10.1016/j.bbagen.2015.05.021.

4. Goedert M, Jakes R, Spillantini MG, Hasegawa M, Smith MJ, Crowther RA. Assembly of microtubule-associated protein tau into Alzheimer-like filaments induced by sulphated glycosaminoglycans. Nature. 1996;383(6600):550-3. doi:10.1038/383550a0.

5. Bagchi DP, Yu L, Perlmutter JS, Xu J, Mach RH, Tu Z et al. Binding of the radioligand SIL23 to alpha-synuclein fibrils in Parkinson disease brain tissue establishes feasibility and screening approaches for developing a Parkinson disease imaging agent. PLoS One. 2013;8(2):e55031. doi:10.1371/journal.pone.0055031.

6. Hudson SA, Ecroyd H, Kee TW, Carver JA. The thioflavin T fluorescence assay for amyloid fibril detection can be biased by the presence of exogenous compounds. FEBS J. 2009;276(20):5960-72. doi:10.1111/j.1742-4658.2009.07307.x.

7. Zhang X, Tian Y, Li Z, Tian X, Sun H, Liu H et al. Design and synthesis of curcumin analogues for in vivo fluorescence imaging and inhibiting copper-induced cross-linking of amyloid beta species in Alzheimer's disease. J Am Chem Soc. 2013;135(44):16397-409. doi:10.1021/ja405239v.

8. Auld DS, Farmen MW, Kahl SD, Kriauciunas A, McKnight KL, Montrose C et al. Receptor Binding Assays for HTS and Drug Discovery. In: Sittampalam GS, Grossman A, Brimacombe K, Arkin M, Auld D, Austin CP et al., editors. Assay Guidance Manual. Bethesda (MD)2004.

9. Paxinos G, Watson CR, Emson PC. AChE-stained horizontal sections of the rat brain in stereotaxic coordinates. J Neurosci Methods. 1980;3(2):129-49. doi:10.1016/0165-0270(80)90021-7.

10. Ma Y, Hof PR, Grant SC, Blackband SJ, Bennett R, Slatest L et al. A three-dimensional digital atlas database of the adult C57BL/6J mouse brain by magnetic resonance microscopy. Neuroscience. 2005;135(4):1203-15. doi:10.1016/j.neuroscience.2005.07.014.

11. Mirrione MM, Schiffer WK, Fowler JS, Alexoff DL, Dewey SL, Tsirka SE. A novel approach for imaging brain-behavior relationships in mice reveals unexpected metabolic patterns during seizures in the absence of tissue plasminogen activator. Neuroimage. 2007;38(1):34-42. doi:10.1016/j.neuroimage.2007.06.032.

12. Schiffer WK, Mirrione MM, Biegon A, Alexoff DL, Patel V, Dewey SL. Serial microPET measures of the metabolic reaction to a microdialysis probe implant. J Neurosci Methods. 2006;155(2):272-84. doi:10.1016/j.jneumeth.2006.01.027.
